# Development of an Effective Corruption-Related Scenario-Based Testing Approach for Robustness Verification and Enhancement of Perception Systems in Autonomous Driving

**DOI:** 10.3390/s24010301

**Published:** 2024-01-04

**Authors:** Huang Hsiang, Yung-Yuan Chen

**Affiliations:** Department of Electrical Engineering, National Taipei University, New Taipei City 23741, Taiwan; s810576103@webmail.ntpu.edu.tw

**Keywords:** autonomous driving, corruption factors, perception system, robustness verification, scenario-based testing

## Abstract

Given that sensor-based perception systems are utilized in autonomous vehicle applications, it is essential to validate such systems to ensure their robustness before they are deployed. In this study, we propose a comprehensive simulation-based process to verify and enhance the robustness of sensor-based perception systems in relation to corruption. Firstly, we introduce a methodology and scenario-based corruption generation tool for creating a variety of simulated test scenarios. These scenarios can effectively mimic real-world traffic environments, with a focus on corruption types that are related to safety concerns. An effective corruption similarity filtering algorithm is then proposed to eliminate corruption types with high similarity and identify representative corruption types that encompass all considered corruption types. As a result, we can create efficient test scenarios for corruption-related robustness with reduced testing time and comprehensive scenario coverage. Subsequently, we conduct vulnerability analysis on object detection models to identify weaknesses and create an effective training dataset for enhancing model vulnerability. This improves the object detection models’ tolerance to weather and noise-related corruptions, ultimately enhancing the robustness of the perception system. We use case studies to demonstrate the feasibility and effectiveness of the proposed procedures for verifying and enhancing robustness. Furthermore, we investigate the impact of various “similarity overlap threshold” parameter settings on scenario coverage, effectiveness, scenario complexity (size of training and testing datasets), and time costs.

## 1. Introduction

In recent years, as major automakers have actively pursued the development of autonomous vehicles, the requirements for designing and testing self-driving cars have become increasingly stringent. These vehicles must demonstrate their reliability and safety to meet the safety standards for autonomous driving. Autonomous driving systems primarily consist of four processing modules: sensor-based perception, localization, trajectory planning, and control. These modules are interdependent, and the performance of the sensor-based perception system is particularly crucial. Insufficient robustness in this system could result in serious traffic accidents and pose significant risks. The sensor-based perception system comprises the primary sensors such as the camera, LiDAR, and radar. These various sensors can detect environmental cues, but each possesses its own strengths and weaknesses. The perception functionality could be susceptible to corruption or misjudgment in different road situations, weather conditions, and traffic environments. Therefore, ensuring the safe and reliable operation of the autonomous perception system in various driving scenarios is of utmost importance as it will directly impact the reliability and safety performance of the self-driving systems [1]. The test coverage impacts the quality of testing for autonomous driving systems. Increasing the coverage of rare driving situations and corner cases is, thus, crucial. To collect data from all possible scenarios through real-world testing would require a minimum of 275 million miles of testing mileage [2], incurring substantial costs and time, rendering it a challenging and economically impractical undertaking.

The testing and verification methods for autonomous driving systems primarily consist of simulation and real-world testing. Currently, we observe that numerous leading automakers are conducting real-world road tests with autonomous vehicles. However, most of these tests are conducted in common and typical scenarios. To test for safety-critical or rare, low-probability adverse driving scenarios and corner cases, one must address specific risks, substantial costs, and time constraints. Even when there is an opportunity to test rare combinations of factors such as weather, lighting, traffic conditions, and sensor noise corruption that lead to failures in autonomous perception systems [3,4,5], replicating the same real-world testing conditions remains challenging and impractical.

On the other hand, the vehicle simulation approach allows for the rapid creation of various test scenarios, including adjustments to different weather conditions within the same test scenario. The advantages of simulation include the ability to easily create the desired scenarios and perform the scenario-based testing at a lower cost and in a safe manner. Currently, there are several vehicle simulation tools available, including Vires Virtual Test Drive (VTD) [6], IPG Automotive CarMaker [7], CARLA [8], etc. The accuracy of the test results depends on how well sensors, vehicles, and environments are simulated. For example, simulating rainfall involves adjusting parameters of the rainfall model, such as intensity. The challenge lies in accurately replicating real-world rainfall conditions, such as an hourly rainfall rate of 20 mm/h, within the simulation environment. Furthermore, the accuracy of modeling simulations for cameras installed behind the windshield of autonomous vehicles is constrained by factors like raindrops impacting the camera’s view, which cannot be completely replicated at present.

In the context of autonomous driving perception systems, object detection is crucial. Any failure or anomaly in object detection can have a significant impact on the system’s prediction and decision-making processes, potentially leading to hazardous situations. Therefore, extensive testing and verification must be conducted under various combinations of conditions. According to ISO 26262 standards [9] autonomous driving systems are permitted a maximum of one failure per $10^{9}$ kilometer driven, which makes it impractical to conduct real-world testing and verification [10].

Currently, there are several real traffic image datasets available, such as nuScenes [11], BDD100K [12], Cityscapes [13], and KITTI [14], which are used to train and validate object detection models. However, these datasets still cannot encompass all potential environmental influences. Some image datasets are only recorded under favorable weather conditions. Moreover, the scenario parameters cannot be quantified to understand the severity of corruption types, including weather-related and noise-related factors, and their impact on object detection capability.

Adverse weather conditions can lead to a decrease in the accuracy of object detection. In addition, when camera noise appears in the image, even a single-pixel corruption has the potential to induce errors or failures in the object detection [15]. This, in turn, can lead to incorrect judgments during autonomous vehicle operation. Presently, there is no comprehensive and quantifiable benchmark test dataset for adverse weather conditions and image noise corruption. Such a dataset could offer sufficient test coverage for verifying the performance of the autonomous driving systems.

While there has been research using vehicle simulation environments to test and verify advanced driver-assistance systems by adjusting various weather, lighting, and camera conditions, there is still a lack of simulation for scenarios where raindrops interfere with the image lens mounted behind the windshield and the severity of the effect of image lens corruption on image quality. In real-world scenarios, this can impact the accuracy of object detection. Therefore, exploring the benefits of simulation and creating effective benchmark test datasets for testing and verification can enhance the reliability and robustness of autonomous vehicle perception systems. This is a topic worth investigating.

This study primarily focuses on integrating raindrops falling on the windshield and various types of weather-related and noise-related corruptions into scenarios within the simulation environment. By adjusting weather-related and noise-related parameters, the aim is to create a comprehensive benchmark test dataset that can assist in testing and validating the reliability and robustness of object detection models. Furthermore, through this benchmark test dataset, the study aims to analyze the vulnerabilities of object detection models. This analysis will help designers identify areas for improvement and propose enhanced training datasets for transfer learning, thereby improving model robustness and reliability.

The primary challenge here lies in the complexity of scenarios involving individual corruption factors and combinations of multiple corruption factors, resulting in a significant amount of test data and time required for verification. To expedite testing and verification in the initial stages of system development, while maintaining the quality of the test dataset, this study presents a corruption similarity analysis algorithm. This algorithm explores the similarity of different corruptions and relies on setting overlapping threshold values to reduce corruption types with higher similarity. The selection of the overlapping threshold value impacts the number of retained corruption types, the size of training and test datasets, training and verification time, and test coverage. By analyzing these factors, an appropriate “corruption overlapping threshold” can be established to obtain an optimal benchmark test dataset that meets the requirements of time cost and test scenario coverage, considering both cost-effectiveness and testing quality.

The remaining paper is organized as follows. In Section 2, the related works are summarized. An effective methodology for generating simulation-based corruption-related testing scenarios to verify and enhance the robustness of perception systems is proposed in Section 3. Subsequently, we analyze and discuss the experimental results in Section 4. The conclusions are presented in Section 5.

## 2. Related Works

The actual road testing and validation of autonomous vehicles may require covering several million kilometers to gather performance statistics, which is primarily relevant to mechanical configurations and algorithm parameters. However, this approach is less effective during the development process, such as the V model development workflow [16]. Relying solely on the total mileage driven for the validation of autonomous driving is an unacceptable solution [17]. Therefore, relying solely on real road testing for validation is almost impractical.

Any failure in testing autonomous driving assistance systems (ADAS) has the potential to compromise safety and lead to unfortunate incidents. Therefore, ref. [18] proposed the integration of multiple ADAS sensors and their corresponding parameters into a virtual simulation platform. The primary contribution lies in the ability to parameterize and adjust the testing and validation of various specific sensors and different mounting positions, facilitating the assessment of sensor fusion algorithms. However, this approach does not delve into the interference caused by varying levels of weather severity or image noise, which could potentially result in sensor failures or misjudgments.

Ref. [19] presented a keyword-based scene description methodology, enabling the conversion of relevant data formats for simulation environments. This transformation facilitates the transition to data formats such as OpenDRIVE and OpenSCENARIO, providing a more efficient means of creating diverse testing scenarios. Nevertheless, there is a relatively limited exploration of the analysis of various weather interference types and different levels of severity within scenarios.

Paper [4] introduced the concept of autonomous driving image perception systems, and developed a tool which can transform normal real-world images into images affected by interference. However, generating a huge number of scenarios and creating more complex image variations using their method can be highly time-consuming. Moreover, a comprehensive analysis of the effect of weather interference on testing and validation is still an area that requires further research.

The primary contribution of paper [10] lies in the establishment of models based on real-world snowfall and foggy conditions. These models enable the creation of image datasets with adjustable parameters, thus enhancing the testing coverage by introducing adverse weather conditions like snowfall and fog. Furthermore, it facilitates the quantifiable adjustment of parameters to create high-interference weather image datasets, aiding in the training and validation of object detection models.

The camera noise in images may lead to errors or failures in AI-based object detection systems [20], prompting numerous scholars to propose various algorithms to detect and eliminate noise in images [21,22]. Furthermore, most of these algorithms rely on common image quality metrics such as the Mean Square Error (MSE), Peak Signal to Noise Ratio (PSNR), and Structural Similarity Indexing Method (SSIM) [22,23] to assess the effectiveness of denoising algorithms. The studies above lacked discussion whether the restored images were sufficient to improve the detection rates of AI object detection systems and the impact of noise removal on the subsequent object detection process.

Ref. [24] employed two different raindrop detection algorithms to calculate the position of raindrops on the windshield and utilized a raindrop removal algorithm to ensure the continued perception capabilities of ADAS, even in the presence of raindrop interference. Ref. [25] examined a vision-based driver assistance system that performs well under clear weather conditions but experiences a significant drop in reliability and robustness during rainy weather. Raindrops tend to accumulate on the vehicle’s windshield, leading to failures in camera-based ADAS. To obtain a real dataset with raindrops on the windshield, paper [26] artificially applied water to a glass surface and placed it 3 to 5 cm in front of a camera. Since the dataset was captured in the real world, it accurately represented light reflection and refraction. However, this method is only suitable for static scenes as each scene requires two images: one with the water-covered glass and another without. Additionally, this method cannot precisely control the size and quantity of water droplets on the glass.

Another raindrop simulation approach presented in paper [27] produced nearly photorealistic results. However, creating scenes and rendering raindrops in this way is time-consuming, taking three minutes for each KITTI image. To be practical, it is necessary to have a method to expedite this process. The authors in reference [28] utilized publicly available datasets with label files (e.g., BDD, Cityscapes) and presented dataset images on a high-resolution computer screen. They placed a 20-degree tilted glass between a high-resolution DSLR camera and the computer screen to simulate a real car’s windshield, but quantifying the raindrops is challenging. Utilizing screen-capture from a camera may result in color distortion and diminish dataset quality.

Reference [13] provided a dataset for training the perception system of autonomous vehicles. However, it contains minor labeling inaccuracies, such as classifying wet roads as rainy weather without distinguishing the severity of rainfall. Training models with inaccurate labels can lead to errors in decision making. As a result, it is necessary to correct the labeling errors before using it. Paper [29] primarily focused on reconstructing real-world scene datasets using a simulation platform. It demonstrated how to parameterize different sensors for simulation matching. Finally, it evaluated the detection results using object detection algorithms with AP and IoU threshold indicators. This comparison involved real data from the front camera module of the nuScenes dataset and synthetic front camera images generated through the IPG Carmaker simulation environment.

In papers [30], the utilization of a game engine for simulation enabled adjustments to various weather conditions. However, these weather settings lacked quantifiable parameters that correspond to real-world environments. Furthermore, the dynamic vehicle simulation in the game engine falls short of realism compared to specialized software tools like VTD (version 2022.4) or PreScan (version 2022.1). Furthermore, it does not support the simulation of diverse sensors and provides only limited image-related data, which may somewhat limit robustness verification.

Reference [31] presented a novel image transformation algorithm capable of adding or removing rain-induced artifacts, simulating driving scenarios in transitioning from clear to rainy weather. However, this dataset lacked image interference caused by raindrops on car windshields and could not quantify the adjustments to different raindrop parameters, somewhat limiting its functionality.

Reference [32] proposed two benchmark test datasets encompassing 15 different image interference scenarios. These datasets involved object displacement and subtle image adjustments, serving to test the resilience of AI classifier models. While these benchmark test datasets covered a variety of interferences, the paper did not account for the similarity and overlap of corruptions between test scenarios. This oversight may lead to ineffective testing and inadequate coverage, resulting in incomplete verification results. Hence, future benchmark test datasets must consider the issue of interference similarity and overlap among test scenarios to ensure both coverage and effectiveness in verification testing.

Paper [33] argued that most autonomous driving data collection and training occurred in specific environments. When faced with uncertain or adverse conditions such as snow, rain, or fog, there are considerable challenges. While there are some available datasets for evaluating self-driving perception systems, there is still a need for more comprehensive and versatile test datasets to continually enhance the overall functionality and performance of these systems.

Paper [34] presented an approach to train AI object detection models using virtual image datasets. The experiments demonstrate that by combining virtual image datasets with a small fraction of real image data, models can be trained to surpass models trained solely with real image datasets. However, this paper did not address the use of virtual datasets augmented with weather or noise interference for training.

In summary, due to the diversity of interference types and varying severity levels, testing all possible interference types and their severity would result in a vast amount of test data and time requirements. There is a need to propose an efficient way to reduce the number of similar interference types to be investigated while maintaining the required test coverage and shortening the testing time effectively.

## 3. Methodology of Simulation-Based Corruption-Related Testing Scenario Benchmark Generation for Robustness Verification and Enhancement

In the context of sensor-based perception systems, we propose a comprehensive method for verifying and enhancing robustness. Our aim is to create a benchmark dataset related to corruption which can be used to verify and improve the robustness of perception systems. We propose a method for generating corruption-related testing scenarios and a simulation toolset to create various scenarios that resemble real traffic environments. We demonstrate that the proposed robustness testing and validation method, constructed with key adverse weather and noise-related corruptions, can increase testing scenario coverage and effectiveness with fewer testing resources. Importantly, the robustness of sensor perception systems can be evaluated using benchmark datasets for testing robustness to ensure the capability and resilience of the perception system.

**Figure 1:**
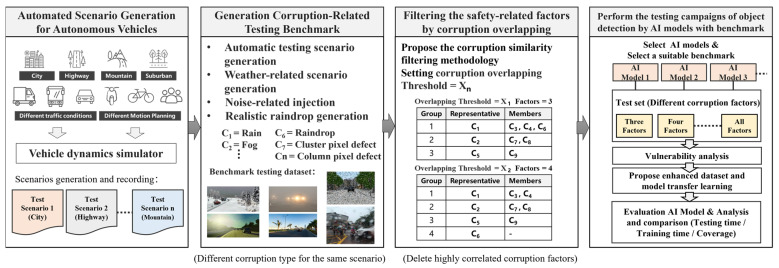
The process of generating benchmark datasets for robustness validation and enhancement of object detection models.

To accelerate the creation of benchmark datasets, we have developed a toolset for generating scenario-based testing benchmarks. This toolset includes weather-related testing scenario generators and sensor noise injectors. Secondly, if robustness testing fails to meet safety requirements, we conduct a vulnerability analysis to identify an improved training dataset that enhances the tolerance of object detection models to weather and noise-related corruptions, thereby improving the robustness of the perception system. We utilize case studies to illustrate the process of creating benchmark datasets for weather-related conditions (such as rain and fog) and noise testing scenarios (such as camera pixel noise). We perform robustness testing on the perception system using object detection models on the benchmark dataset and then analyze the vulnerabilities of the object detection models. Conversely, the growing number of corruption types being considered will lead to a rapid increase in testing and validation time. To ensure the quality of benchmark datasets and facilitate rapid testing and validation in the initial phases of system development, this study also proposes a corruption similarity analysis algorithm. The goal of the algorithm is to efficiently filter similar types of corruption while maintaining high testing coverage. This is achieved by compressing the benchmark dataset to reduce the testing and validation time. To achieve this goal, we will analyze the effect of corruption overlapping threshold settings on the number of representative corruption types, testing scenario coverage and effectiveness, and benchmark complexity and testing time cost. Next, we will demonstrate the process of enhancement by employing data augmentation techniques with efficient training datasets to improve the robustness of object detection models. Finally, we will validate the feasibility and effectiveness of the proposed process for verifying and enhancing robustness. The process of generating benchmark datasets related to corruption for validating the robustness and improving object detection models is illustrated in Figure 1 and described in the following subsections.

### 3.1. Automated Test Scenario Generation

The perception system of autonomous vehicles encounters a variety of road scenarios, including city, mountainous, rural, and highway environments. These scenarios involve various types of traffic participants, such as vehicles, pedestrians, motorcycles, and bicycles. In this study, we utilize the Vires VTD simulation tool, which is specifically designed for the development and testing of autonomous vehicles and ADAS. This tool enables the simulation of various sensors and the adjustment of relevant sensor parameters. By creating diverse road scenarios, including various types of traffic participants, and simulating different weather conditions, we can quickly generate a large number of traffic scenarios. This will provide a dataset with a wide range of scenarios for testing and validation purposes.

Furthermore, in comparison to real-world testing and validation on actual roads, vehicle simulation software not only facilitates the creation and recording of various driving scenarios but also permits the repetitive reproduction of the same scenarios for testing and validation purposes. It ensures that all traffic participants and vehicle movement paths remain consistent, with variations limited to safety-related weather and sensor noise corruptions. This is beneficial for testing and validating the perception system of autonomous vehicles, enabling a comparative analysis of the impact of these corruptions on the system’s robustness.

**Figure 2:**
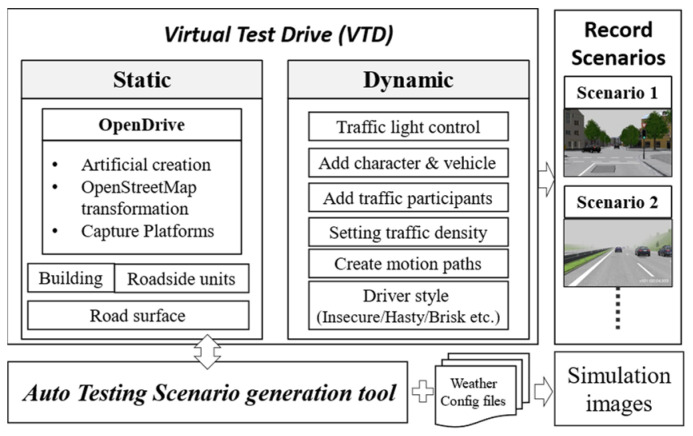
An overview of the automated testing scenario generation process using VTD.

As depicted in Figure 2, the process commences with the setup of scenes in the Vires VTD simulation platform, comprising two primary components: static and dynamic configurations. The static configuration involves constructing an OpenDrive map, placing buildings and roadside elements, and designing road surfaces. The OpenDrive map can be created by manually drawing it or by using open-source OpenStreetMap data with specific area selections for conversion. For more specialized high-definition maps, professional mapping tools are required for scanning and measurement. The dynamic configuration includes adjusting traffic light settings, adding vehicles or other traffic participants, modifying traffic, planning routes, and adapting driving styles. Dynamic configuration enables the realization of various critical scenarios or hazardous situations. These test scenarios can be saved in the OpenScenario format.

Additionally, the road scenes and test scenarios created through VTD support both OpenDrive and OpenScenario formats, which are two standard formats commonly used for exchanging information between simulation tools. Therefore, the identical test scenarios can be imported into other simulation tools that support these standard formats, making it easier to collaborate on testing and validation using different tools.

Every time we run the VTD simulation, even if we set a fixed traffic density, the flow of traffic and the behavior of traffic participants are generated randomly. This variability leads to different vehicle positions, types, and movement paths in each simulation run. To ensure consistency in each simulation run, we use a scene recording approach to capture every unique test scenario and traffic participant. This allows us to precisely replay these scenarios, ensuring the reproducibility of each test.

**Figure 3:**
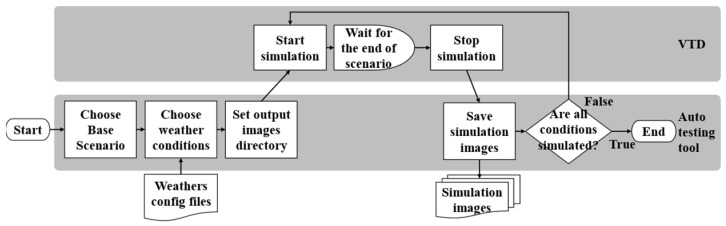
Workflow diagram of the automated testing scenario generation tool.

As shown in Figure 3, this research has developed an automated test scenario generation tool to facilitate the automated generation of various test scenarios and simulate different weather conditions. This tool is intended to be used in conjunction with the Vires VTD simulation software (version 2022.4). Users only need to select a base scenario from a list of previously recorded test scenarios and configure relevant weather parameters in the weather configuration file. Each test scenario includes detailed traffic environment information, including weather conditions. Finally, users have the option to specify the output directory for the generated image dataset. VTD can simulate the base scenario based on the weather configuration file, rapidly generating image datasets for different test scenarios with various weather conditions.

### 3.2. Test Benchmark Dataset Generation

Self-driving perception systems encounter numerous real-world challenges, such as diverse traffic conditions and varying levels of interference, primarily classified as weather-related environmental corruption and sensor noise interference. When the object detection models of self-driving perception systems are exposed to different types and levels of corruption, it can impact the reliability and robustness of these models. Therefore, in this study, we utilize the Vires VTD simulation tool along with an automated test scenario generation tool to create a dataset of weather-related corruption, including raindrop corruption on the vehicle’s windshield and camera noise corruption images. This approach enables us to generate a benchmark test dataset containing multiple types of corruption in different scenarios.

#### 3.2.1. Weather-Related Corruptions

In real driving scenarios, the most common weather-related environmental corruptions are rain and fog. These corruptions are typically characterized by units of rainfall intensity and visibility to represent different levels of severity. However, many image datasets do not explicitly distinguish between these factors or only categorize rainy conditions into a few severity levels. But how can we align these severity levels with actual units of rainfall intensity to ensure that the selected severity ranges meet the requirements? In real-life scenarios, heavy rain can have a significantly more severe impact than light rain. If the entire benchmark dataset only captures instances of light rain, evaluating the robustness and reliability of object detection models under rainy conditions may lack objectivity. Even if the test results show high accuracy, we still cannot guarantee the perception system’s performance in heavy rain, which could pose potential hazards.

On the other hand, during actual rainy and foggy weather, the intensity of rainfall and visibility are not constant; they often vary within a range. Therefore, it is advantageous to establish distinct severity levels for each type of corruption, as illustrated in Table 1. For instance, in the case of fog, the visibility in region 1 (R_1_) ranges from 200 to 164 m, and for rain, the rainfall intensity is between 43 and 48 mm/h in R_1_. Each of these types of corruption within R_1_ has five subsections, as shown in Table 2. By establishing a test benchmark in this manner, the object detection capability can be tested and verified more comprehensively under various types of corruption and severity levels.

The relevant weather configuration parameters in the VTD (version 2022.4) simulation tool are presented in Table 3. To facilitate user convenience, we have imported these parameters into our developed automated test scenario generation tool in .CSV format. This feature enables users to easily edit and adjust the relevant weather parameters using Excel or other software. These weather-related parameters include precipitation type, precipitation intensity, visibility, sky condition, and time of occurrence. In the VTD rain and snow settings, there is no option to adjust the intensity. Only different density parameters from 0.0 to 1.0 can be set, which affects the density of rain and snow in the VTD simulated images.

**Figure 4:**
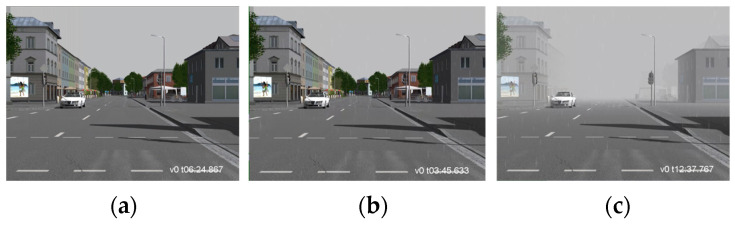
VTD environmental simulation of different weather conditions. (**a**) No rain; (**b**) rain; (**c**) rain with visibility influence.

Figure 4a,b show image frames from the VTD simulation without rain and with rain, respectively. By analyzing these image frames alone, the differences in corruption are not very noticeable, except for the presence of raindrops. The main reason for this is that in real-world rainy conditions, there is not only the influence of raindrops but also a decrease in visibility. To better simulate real-world rainy conditions, we utilized the formula suggested in [35,36,37] to calculate the visibility corresponding to a specific rainfall rate. When the rainfall rate exceeds 7.6 mm/h, you can calculate the corresponding visibility using Equation (1). This will show the relationship between different rainfall rates, where “rainfall rate” represents the amount of rainfall and “visibility” represents the distance one can see clearly. In this way, you can set rainfall parameters similar to those in Table 1 and calculate the corresponding visibility in rain. For example, for rainfall rates of 43~48 mm/h, the corresponding visibility is approximately 300~200 m, as shown in Table 4. Figure 4b shows simulating rain alone, without considering the visibility effect of rain in VTD, compared to Figure 4c which calculates corresponding visibilities for different rain rates. Clearly, the simulated rainy image frames with visibility influence closely resemble real rainy scenarios. This method enables the quantification of parameter adjustments for different severity levels, allowing users to quickly configure various weather parameters and test scenarios. When combined with the automated test scenario generation tool, it becomes possible to efficiently generate more realistic weather-related scenario-based test benchmarks using the VTD simulation tool.

(1) $${\mathrm{Vis}}_{R}=-863.26RP_{R}{}^{0.003}+874.19$$

#### 3.2.2. Noise-Related Corruptions

In addition to being affected by weather-related factors that impact reliability and robustness, self-driving perception systems may also be susceptible to various image noise corruptions. Examples of image noise corruptions include single-pixel defects, column pixel defects, and cluster pixel defects, which have the potential to cause object detection failures. To enable thorough testing and validation, we have created a tool for injecting image noise. This tool enables the quantification of parameters to adjust various types of image noise and corruption percentages, thereby generating image corruptions of different severities.

Furthermore, in addition to the image noise caused by the camera itself, some cameras used in self-driving perception systems or advanced driver assistance systems are mounted behind the vehicle’s windshield. Consequently, when vehicles operate in rainy conditions, numerous raindrops adhere to the windshield, obstructing the camera’s view. The size of these raindrops varies, and the density of raindrops varies with different rainfall rates. Examining the impact of raindrop interference on the reliability and robustness of object detection in varying levels of rainfall is a key focus of this research.

**Figure 5:**
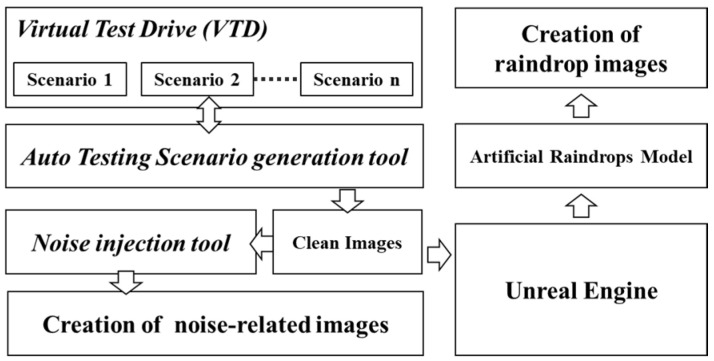
Workflow for generating noise-related and raindrop images.

As illustrated in Figure 5, we utilize the VTD simulation platform along with an automated test scenario generation tool to produce image frames free from weather corruption. Subsequently, we input the original clean image datasets into our developed image noise injection tool, as well as an artificial raindrop generation tool built on the Unreal Engine platform. This enables us to create image datasets with measurable variations in corruption severity or different raindrop sizes and densities within the same test scenarios.

**Figure 6:**
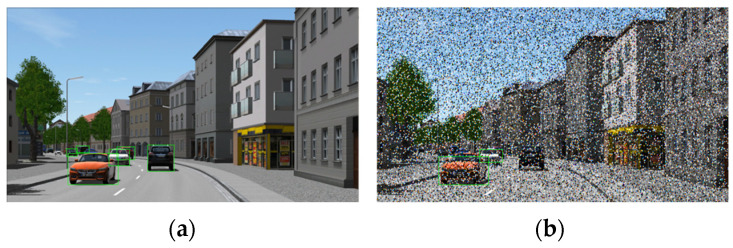
Comparative analysis of the impact of noise corruption on object detection models in images: (**a**) no corruption, (**b**) noise corruption.

Figure 6a displays an image frame with clear blue skies and no image noise corruption, where the object detection model accurately identifies and labels each vehicle object. However, when there is image noise corruption and the same object detection model is used, it is evident that several vehicles cannot be successfully identified. This could potentially lead to a failure in the self-driving perception system, as depicted in Figure 6b. Conducting a significant number of tests with various levels of camera image noise corruption during real-world autonomous driving validation is challenging because it does not allow for controlled adjustments of different image corruption types and severity levels. Therefore, in order to improve the test coverage of autonomous driving perception systems, it is essential to be able to test and analyze the robustness and reliability of object detection models under various image noise corruption conditions.

**Figure 7:**
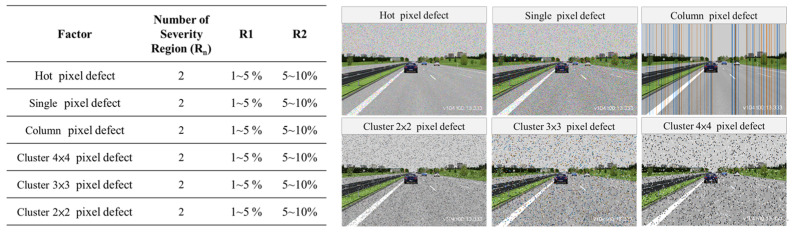
Corruption percentage for different severity levels of noise corruption types.

To simplify the process of generating image noise corruption, users only need to configure the original clean image data and specify the type of image noise to be injected. Users can also specify various levels of image noise corruption. Once this configuration is prepared, it can be imported into the image noise injection tool. The tool randomly selects pixel positions in the images according to the specified corruption type and percentage, and then injects noise into those positions. It ensures that the pixel errors meet the specified percentage requirements, enabling the rapid generation of diverse image noise datasets. Figure 7 illustrates the configuration parameters for different types of image noise corruption and the severity regions. The six types of image noise corruption include Hot pixel defect, Single pixel defect, Column pixel defect, Cluster 2 × 2 pixel defect, Cluster 3 × 3 pixel defect, and Cluster 4 × 4 pixel defect.

#### 3.2.3. Raindrop Factor

To achieve a more realistic effect of raindrops hitting the windshield and interfering with the camera’s image, we must consider the impact of different rainfall intensities and raindrop properties [35]. The impact of corruption caused by raindrops on the camera installed behind the vehicle’s windshield primarily depends on three parameters: raindrop density, raindrop size, and raindrop velocity. However, the most significant factor influencing these three parameters is the intensity of rainfall.

Assuming there is a 10-cubic meter structure, the rate of rainwater accumulation varies under different rainfall intensities. We can use Formula (2) to calculate the volume of rainwater that can accumulate in the cube in one hour for different rainfall intensities. In the formula, CV represents the hourly accumulated volume of rainwater in the cube (in milliliters), HR represents the rainfall intensity (in mm/h), and L represents the side length of the cube (in meters).

Next, based on the research findings from references [24,25,26], we estimate the approximate diameters of raindrops corresponding to different rainfall intensities. When the intensity of rainfall is greater, the diameters of raindrops tend to be larger. Different raindrop diameters not only affect the volume of the raindrops but also influence the speed at which they fall [36]. Assuming the constant rainwater capacity in the cube, the diameter of raindrops will impact the total number of raindrop particles that accumulate in one hour. Additionally, the speed at which raindrops fall will impact the number of raindrop particles that interfere with each camera image.

Assuming a raindrop falling from a height of 20 m, we can calculate the raindrop’s velocity relative to different raindrop diameters using Formula (3) proposed in paper [36], where *V* represents the raindrop’s velocity, and *D* represents the raindrop’s diameter.

(2) $$\mathrm{CV}=(L\times {100)}^{2}\times \frac{HR}{10}$$

(3) $$V=9.5\left[1-\exp(-6D)\right]$$

To calculate the rainwater capacity accumulated in a 10-cubic meter structure within one hour, along with the corresponding raindrop diameters and the number of raindrop particles, we first use Formula (4) to compute the volume of raindrops associated with their diameters. In this formula, *RV* represents the raindrop volume, and *D* represents the raindrop diameter. Next, we can use Formula (5) to estimate approximately how many raindrops fall into the cubic structure per hour.

(4) $$RV=\frac{4}{3}\pi \times {\left(\frac{D}{2}\right)}^{3}$$

(5) $$CP=CV\times \frac{1000}{RV}$$

The distance between the camera installed behind the windshield of the vehicle affects the number of raindrops captured in the image when the raindrop density remains constant. When the camera is positioned farther away from the vehicle’s windshield, more raindrops will be captured in the image. Conversely, if the camera is closer to the windshield, fewer raindrops will be captured in the image. Therefore, to accurately capture raindrop corruption in the camera’s image, we must consider the distance between the camera and the vehicle’s windshield.

However, the camera installed behind the vehicle’s windshield captures only a small portion of the total area of the windshield. The actual size of the windshield area captured by the camera depends on the distance between the camera and the windshield. This area is significantly smaller than the area of the 10 m cube. The number of raindrops falling on the portion of the windshield captured by the camera in one second can be calculated by Expression (6). Then, based on the camera’s frame rate (fps), we can calculate the average number of raindrops landing in each frame using Equation (7). Consequently, we can attain a more realistic representation of raindrop corruption in the simulation experiments.

(6) $$RW=\frac{area\;of\;windshield}{bottom\;area\;of\;cube}\times \frac{CP}{3600}$$

(7) $$Raindrops/frame=RW\;÷fps\;\left(camera\right)$$

**Figure 8:**
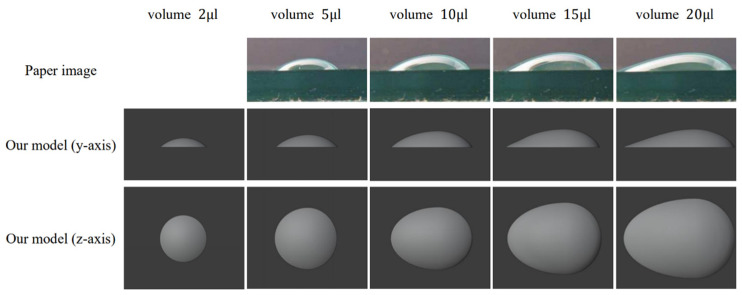
Comparison between real raindrops [37] and simulated raindrop models.

Raindrops of different sizes result in unique shapes when they land on the windshield. To create more realistic raindrop shapes, we drew inspiration from the diverse shapes of real raindrops as described in paper [37], and we developed raindrop models specifically suited to our simulated environment, as shown in Figure 8. The strong resemblance between our simulated raindrop models and actual raindrops is apparent in Figure 8.

Table 5 outlines the range of raindrop volumes covered by different raindrop models. However, it is important to note that within the same model, different raindrop sizes may not have the same shapes. First, we select a suitable raindrop model based on raindrop volume, as outlined in Table 5. Then, we use Formula (8) to compute the scaling factor of the model, which allows us to adjust the size of the raindrops by zooming in or out. In this formula, “*Raindrop_volume_*” represents the volume size of the raindrop, and “*Model_volume_*” represents the volume of the selected raindrop model. This approach allows us to adjust the raindrop models within the UE4 simulation platform to generate different raindrop sizes that closely mimic real raindrop shapes. This enhances the reliability of our testing and validation.

(8) $$Model_{scale}=\sqrt[{3}]{Raindrop_{volume}÷Model_{volume}}$$

In a real rainfall scenario, as more raindrops fall on the windshield of a car, the initially stationary raindrops on the windshield may merge with other raindrops. When they reach a certain size, they will flow down the windshield instead of remaining in a fixed position. Therefore, in our raindrop generation, we have integrated a continuous model to calculate the size of each raindrop as it coalesces on the windshield. When the raindrops reach a size sufficient to flow down the windshield, we capture the image frames of raindrops descending. Through this tool, we can generate a dataset of raindrop-corrupted images corresponding to various rainfall intensities.

In contrast to the raindrop generation tool proposed in [37], our method utilizes the UE4 simulation platform for raindrop generation and takes approximately 0.833 s per frame to generate raindrop-corrupted images. This signifies a substantial enhancement in the efficiency of creating raindrop-corrupted image datasets compared to the approximately 180 s per frame required by the method outlined in the literature.

### 3.3. Safety-Related Corruption Similarity Filtering Algorithm

Currently, there is no clear specification outlining the testing criteria for the benchmark test set of autonomous vehicle perception systems. Additionally, there is no research team or organization that can guarantee that the test items included in a self-designed benchmark test set encompass all possible corruption factors. Given the highly complex driving environment that autonomous vehicle perception systems face, most studies indicate that a greater number of test items leads to better test coverage [38]. Continuously expanding the test items to include a large number of repetitive or similar testing scenarios can result in excessively long testing times, leading to increased testing complexity and costs. If reinforcement training of object detection models includes numerous repetitive or non-critical scenarios, it not only consumes significant time but also fails to guarantee substantial enhancement of the robustness or overall performance of the object detection model. This can have a significant impact on the development timeline for autonomous driving and waste the time and resources invested.

**Figure 9:**
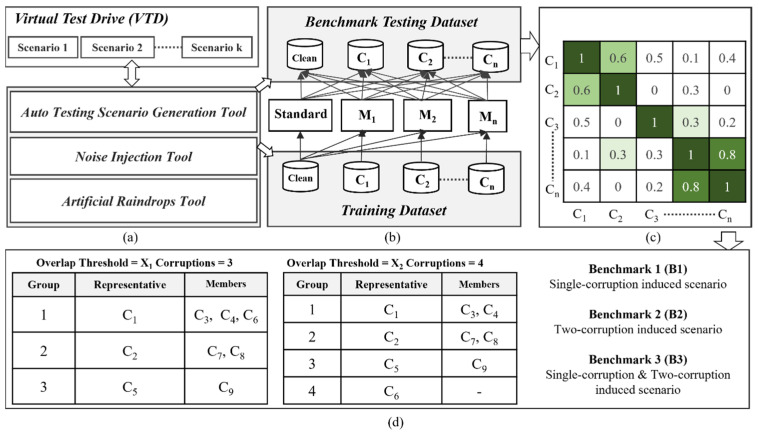
Overlapping analysis procedure and corruption filtering concept among different corruption types. (**a**) Generation of scenario corruption; (**b**) training and benchmark testing datasets; (**c**) “In the corruption overlap score table, the darker the color, the higher the similarity between the two”; (**d**) results of corruption filtering methodology and benchmark types.

In response to the aforementioned issues, this study proposes a safety-related corruption similarity filtering algorithm to effectively decrease the number of test items while maintaining a high level of test coverage. Figure 9 illustrates the procedure for overlapping analysis and the concept of corruption filtering among n types of corruption denoted as C_1_~C_n_. In the beginning, we adopt previously introduced scenario generation methods for weather-related, noise-related, and raindrop corruptions to construct two datasets: a benchmark testing dataset and a training dataset. The establishment process is as follows: we select two sets of corruption-free (or clean) datasets, both originating from the same environmental context, such as a city environment, but with distinct testing scenarios and traffic participants. One clean dataset is used to generate image datasets with n different types of corruption for training purposes, while the other clean dataset is used for benchmark testing, as depicted in Figure 9a,b. The dataset labeled as C_i_, i = 1 to n, as shown in Figure 9b, represents a dataset with C_i_ corruption type.

Subsequently, we selected an AI object detection model and conducted transfer learning using this model along with training datasets containing various corruptions. The number of trained models equals the total number of different types of corruption, plus one standard model trained using a clean dataset without any corruption, referred to as the Standard model. Apart from the Standard model, the other object detection models are trained using a single type of corruption, along with a clean dataset, for transfer learning. Ultimately, we obtain n distinct object detection models trained to detect different types of corruptions, denoted as M_1_~M_n_, in addition to the standard model, as illustrated in Figure 9b.

Figure 9c illustrates the overlap scores between pairs of corruption types. These scores are used to analyze the degree of correlation between two different corruptions, with higher overlap scores indicating a higher degree of similarity between them. We used the overlap score calculation method as proposed in paper [39]. First, we define the robustness score (RS) of model m for corruption type CT, as shown in Equation (9), where *A_clean_* represents the detection accuracy of the model on an uncorrupted test dataset and represents the detection accuracy of the model on the same test dataset corrupted by a specific corruption type *C_T_*. Equation (9) quantifies the model’s robustness performance under a specific type of corruption, where a lower score indicates insufficient robustness of the model to this corruption type, necessitating improvement. Equation (10) provides the formula for calculating the corruption overlap score between two different corruptions, *C*_1_ and *C*_2_. Here, “standard”, “*M*_1_”, and “*M*_2_” represent the selected models using an uncorrupted training dataset and the same training dataset with data augmentation of *C*_1_ and *C*_2_, respectively. The score in Equation (10) ranges from 0 to 1, with a higher score indicating a greater overlap and similarity between *C*_1_ and *C*_2_. According to Equation (10), we can construct a two-dimensional corruption overlap score table, denoted as the overlap score OS(NC, NC), where NC represents the number of corruption types. OS(i, j) represents the overlap score between corruption type i and corruption type j.

(9) $$RS_{C_{T}}^{m}=\frac{A_{C_{T}}}{A_{clean}}$$

(10) $$O_{C_{1},C_{2}}=\max\left\{0,\frac{1}{2}\times \left(\frac{RS_{C_{2}}^{M_{1}}-RS_{C_{2}}^{s\tan dard}}{RS_{C_{2}}^{M_{2}}-RS_{C_{2}}^{s\tan dard}}+\frac{RS_{C_{1}}^{M_{2}}-RS_{C_{1}}^{s\tan dard}}{RS_{C_{1}}^{M_{1}}-RS_{C1}^{s\tan dard}}\right)\right\}$$

Next, we propose an efficient corruption similarity filtering algorithm, as described in Algorithm 1. This algorithm is based on the given overlap score table OS(NC, NC) and an overlap threshold. It aims to identify a minimal set of corruption types where the correlation between these corruption types is less than the specified overlap threshold. Using the filtering algorithm, Figure 9d displays the results of the corruption type after applying the algorithm with NC = 9. For instance, when an overlap threshold is set to X_1_ (where 0 < X_1_ < 1), the algorithm preserves three representative corruption types: C_1_, C_2_, and C_5_. This implies that when the overlap threshold X_1_ is applied, C_1_ can represent C_3_, C_4_, and C_6_ because the overlap scores OS(C_1_, C_3_), OS(C_1_, C_4_), and OS(C_1_, C_6_) are all greater than or equal to the overlap threshold X_1_. Similarly, C_2_ and C_5_ represent two other types of corruption. In this demonstration, nine corruption types are partitioned into three groups, as shown in Figure 9d, and C_1_/C_2_/C_5_ are representative corruption types for group 1/2/3, respectively. The choice of the overlap threshold will affect the number of eliminated corruption types. Clearly, a lower threshold will result in a smaller number of remaining corruption types after the filtering algorithm.
**Algorithm 1:** Corruption Similarity Filtering Algorithm**Input:** given a two-dimensional overlap score table OS(*NC*, *NC*) where *NC* is the number of corruptions and OS(*i*, *j*) represents the overlap score between corruption *i* and corruption *j*. Set up an overlap threshold θ to guide the filtering of corruptions from the overlap score table. **Output:** a subset of *NC* corruptions which will be used to form the benchmark dataset *k* ← *NC*
Based on the overlap score table, for each corruption *i*, *i* = 1 to *k*, we count how many overlap scores between the considered corruption *i* and corruption *j*, *j* = 1 to *k* and *j* ≠ *i*, are greater than or equal to the overlap threshold θ and record the counting number for each corruption *i* in the overlap score table. Therefore, each corruption *i*, *i* = 1 to *k*, has the number that represents the number of corruptions where their overlap scores are greater than or equal to the overlap threshold θ. Moreover, the average overlap score for the corruption *i* can be calculated from the overlap scores that are greater than or equal to the overlap threshold θ.**if** the counting numbers among the considered corruptions in the overlap score table are all zero, **then** go to Step 5**else** we select the corruptions which have the highest counting number among the considered corruptions.**if** the number of corruptions selected in Step 2 is greater than one, **then** {select a corruption which has the largest average overlap score among the selected corruptions derived from Step 2.}The overlap score table is modified according to the following rule: the corruptions are removed from the overlap score table if their overlap scores with the selected corruption are greater than or equal to overlap threshold θ. After removing process, the number of corruptions remained is assigned to variable *k*, and the modified overlap score table becomes OS(*k*, *k*).**if** *k* is one **then** end of the algorithm and the output is the remaining corruption.else go to Step 1End of the algorithm and the output is the OS(*k*, *k*), the remaining *k* corruptions to be used in the benchmark dataset.

**Figure 10:**
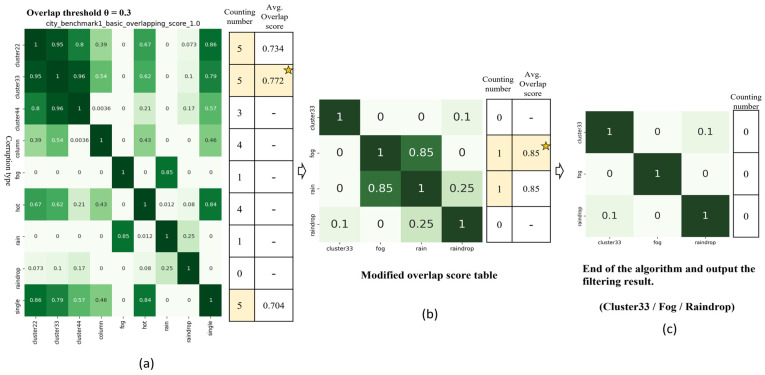
An example of the corruption similarity filtering algorithm. In the corruption overlap score, the darker the color, the higher the similarity between the two. Asterisks represent the corruption types with the highest counting number and the largest average overlap score in that round. (**a**) shows the results of Step 1 for the first round; (**b**) Modified overlap score table and confirm whether the counting numbers for each corruption are all zero; (**c**) The algorithm finished and outputted the filtering result.

An example of the corruption similarity filtering algorithm is illustrated in Figure 10, with a total of nine types of corruptions. Initially, we assume an overlap threshold (θ) of 0.3. The counting number table in Figure 10a shows the results of Step 1 for the first round. In the initial calculation, the highest counting number is 5, attributed to Cluster 22, Cluster 33, and single corruptions, as shown in Figure 10a. As a result, it is necessary to select the corruption which has the largest average overlap score among the selected corruptions. Therefore, Step 3 selected Cluster 33 and removed other corruption types corresponding to Cluster 33 in the table, where their overlap scores are greater than or equal to the overlap threshold. After this modification, the total number of corruptions in the updated overlap score table was adjusted from the original 9 to 4. Next, the modified overlap score table, as illustrated in Figure 10b, was used for another round of calculations to confirm whether the counting numbers for each corruption are all zero. In Step 1 of the second round, the highest counting number is 1, attributed to both fog and rain corruptions, with the average overlap score being the same. Therefore, it is possible to randomly choose one of them as the retained corruption type. In this instance, Fog was selected as the retained corruption for the second round. Consequently, the overlap score table was modified once again, and the updated number of corruptions was adjusted from 4 to 3. Finally, in the third round, we confirmed that the counting number of all corruptions in the modified overlap score table was zero, as shown in counting number table in Figure 10c. As a result, the algorithm finished and outputted the filtering result. A total of three corruptions were retained: Cluster 33, Fog, and Raindrop.

Through this algorithm, we can establish the overlap threshold based on different levels of testing requirements. Then, we can use the representative corruption types after filtering to construct the benchmark testing dataset, as shown in Figure 9d. Figure 9d illustrates the presence of three distinct types of benchmark testing datasets: B1, B2, and B3. Here, B1, B2, and B3 represent scenarios with single corruption, two corruptions, and a combination of single and two corruptions, respectively. The filtering algorithm enables the removal of similar types of corruption, with the main objective being to identify a smaller number of corruption types that represent the original set of corruption types. This can lead to a shorter training and testing time for object detection models and can also be used to assess the quality of testing datasets, ensuring that the required test coverage is achieved within a shorter testing period. It is particularly beneficial during the early stages of perception system design as it accelerates the development process.

The corruption grouping algorithm presented in Algorithm 2 is designed to identify the corruption groups, as shown in Figure 9d. The primary idea behind the corruption grouping algorithm is to utilize the filtered corruption types obtained from the corruption similarity filtering algorithm. For each filtered corruption type, the algorithm aims to discover the other members of the corruption group.
**Algorithm 2:** Corruption Grouping Algorithm**Input:** given a two-dimensional overlap score table OS(*NC*, *NC*) where *NC* is the number of corruptions and OS(*i*, *j*) represents the overlap score between corruption *i* and corruption *j*. Set up an overlap threshold θ. **Output:** corruption groupsAfter running the corruption filtering algorithm under a specific overlap threshold, we can obtain the reduced corruption types (C_1_, C_2_, …, C_k_).For each C_i_, *i* = 1,…*k*, its corruption group can be formed by the following method from the two-dimensional overlap score table.Group forming method: for corruption type C_i_, there are *NC*-1 corruption pairs (C_1_, C_i_), (C_2_, C_i_), … (C_i−1_, C_i_), (C_i+1_, C_i_),… (C_NC_, C_i_). In the *NC*-1 corruption pairs, if its overlap score is greater than or equal to overlap threshold θ, then the corruption type in the pair which is not C_i_ is saved to the C_i_ corruption group. Clearly, we can use the corruption C_i_ to represent the corruption types in the C_i_ corruption group.

By adjusting overlap threshold, the number of retained corruption types after filtering will vary, leading to different testing and validation times for object detection models. Equation (11) can be used to calculate the time required for testing and validation with different benchmark testing datasets. The explanation is as follows: Define *NI* as the total number of image frames in the original corruption-free dataset. Through the corruption filtering algorithm, the number of retained corruption types is denoted as *k*. Corruption types are represented as *C*_i_, where the range of i is from 1 to k. Each corruption type *C*_i_ has a different number of severity regions, defined as *NSR*(*C*_i_). Benchmark 1 (B1) is a single-corruption-induced scenario dataset which is constructed by the following method: We first need to generate single-corruption-induced scenarios for each corruption type and then collect all single-corruption-induced scenarios created for each corruption type to form the B1. Each severity region within a corruption type uses the original corruption-free dataset to generate its single-corruption induced scenarios, and therefore, there are *NSR*(*C*_i_) sets of single-corruption-induced scenarios for corruption type *C*_i_. As a result, we can see that the testing time for each corruption type is the result of multiplying *NSR*(*C*_i_), *NI*, and *DT*_m_. Here, *DT*_m_ is defined as the estimated time it takes for the object detection model m to recognize a single frame, allowing us to calculate the overall testing and validation time *BTT*_m_ using Equation (11).

(11) $$BTTm=\overset{k}{\underset{i=1}{\sum }}NSR\left(Ci\right)\times NI\times DTm$$

### 3.4. Corruption Type Object Detection Model Enhancement Techniques

The robustness of object detection models in autonomous driving perception systems can be enhanced by implementing model transfer learning using datasets that include various corruption types proposed in this study. However, utilizing datasets that contain a wide range of corruption types also results in significant time and cost for model training. After using filtering algorithms to reduce corruption types with high similarity, we can proceed with enhancing the model through training using the remaining corruption types. This approach helps reduce the time required for training model enhancements.

The calculation of the time required for model enhancement training using corruption type image datasets is represented by Equation (12). Here, we define *NEC* as the number of corruption types to be enhanced, and the enhanced corruption types used are denoted as *EC*_i_, where i ranges from 1 to *NEC*. Additionally, for each corruption type, the number of severity regions for each enhanced corruption type is defined as *NER*(*EC*_i_), and the regions for each enhanced corruption type are denoted as *EC*_i_(R_j_), where j ranges from 1 to *NER*(*EC*_i_). The number of images used for model enhancement training is *NEI*.

In Equation (12), the number of images required for each severity region of each corruption type is NEI, and the number of input images for each step of the model enhancement transfer learning is *IS*. The estimated training time for each step of the model transfer learning is *ET*_m_. The total number of epochs for model enhancement transfer learning is NE. Finally, using these parameters, we can calculate the total training time *MET*_m_ required for enhancing the object detection model m.

(12) $$METm=\sum_{i=1}^{NEC}NER(ECi)\times \frac{NEI\times ETm\;\times NE}{IS}$$

Training representatives from each type of corruption within each corruption group are expected to enhance the resilience of each corruption type within the group. As depicted in Table 6, there are two distinct training methods for object detection models. The first approach utilizes the corruption filtering algorithm to eliminate corruption types with high similarity and then utilizes the remaining corruption types to improve the model. The second approach involves training the model using all original types of corruption, which requires the most training time. In the testing dataset, we categorize it into four types. The first type is the original baseline testing dataset, which includes test image data for all types of corruption, resulting in the longest overall testing time. The second approach uses filtered corruption types to represent the original types of corruption, thereby reducing the overall testing time compared to the original baseline testing dataset. The third and fourth types involve using a filtering algorithm to reduce high-similarity corruption types, resulting in the creation of two corruption groups: (Fog and Rain) and (Hot, Single, Cluster 22/33/44). Within these groups, specific types of corruption (e.g., Fog and Cluster 22) represent their respective corruption categories. The concept of filtering aims to decrease the variety of corruption types in order to simplify training and testing. We need to verify the effectiveness of reducing different types of corruption in the training and testing processes. Table 6 presents a comparative analysis of various training datasets for object detection models on different testing datasets. The actual experimental results will be presented in Section 4. Here, we are using this table to illustrate the goals of our approach that we are striving to achieve. In Table 6, the contrast between A and C demonstrates the training effectiveness of filtered corruption types. The smaller gap (between A, C) indicates that our proposed approach can effectively reduce the model training time while maintaining the model quality compared to the model trained with all original types of corruption. Similarly, the difference between A and B indicates the effectiveness of the test dataset constructed using filtered corruption types. The smaller gap (between A, B) indicates that the testing dataset, which is formed by filtered corruption types, can still maintain good testing coverage while significantly reducing testing time. Next, when the value of E approaches G, it indicates that using the fog corruption type to represent the (Fog and Rain) corruption group has a positive impact. Similarly, as the value of F approaches H, it indicates that using the Cluster 22 corruption type to represent the (Hot, Single, Cluster 22, 33, 44) corruption group has a significant impact.

In real-world driving scenarios, it is common for multiple corruption types to occur simultaneously, such as rain and heavy fog. This study aims to analyze and investigate how the simultaneous occurrence of two different corruption types affects the model’s detection performance. It is essential to consider that when dealing with a huge number of corruption types, each having various severity regions and subregions, the resulting dataset can become extensive and complex. As shown in Table 7, we have already applied a filtering algorithm to remove highly similar corruption types and were left with two corruption types: fog and cluster 22. The object detection model has been tested and validated separately for each of these two corruption types. Using the testing data, we can analyze which severity region within a specific corruption type yields the poorest detection performance. This analysis is referred to as the model’s vulnerability analysis. For instance, if the model performs poorly in the fog corruption type’s severity region with visibility ranging from 50 to 20 m, it indicates that this region requires special training to enhance the model’s robustness. Similar vulnerability points can be identified for the cluster 22 corruption type.

Next, we take the vulnerability region of the fog corruption type to determine the midpoint of the visibility region, i.e., 35 m, and redefine two severity subregions: SR1 (visibility ranging from 50 to 35 m) and SR2 (visibility ranging from 35 to 20 m). These two subregions can be used to define the severity setting for combinations of the fog corruption type with other corruption types. We can then create the possible enhanced training datasets for combinations of the fog and cluster 22 corruption types and conduct model transfer learning. Looking at Table 7, for example, the combination of fog and cluster 22 can result in four possibilities: (42.5 m, 13.5%), (42.5 m, 14.5%), (27.5 m, 13.5%), and (27.5 m, 14.5%). This approach is expected to effectively reduce the size of the training dataset for two-corruption-induced scenarios while maintaining good coverage. Training the model with these two-corruption-type combinations can enhance its detection performance when both corruption types occur simultaneously.

As depicted in Table 8, we aim to compare and analyze the robustness performance of four different models on Benchmark 1 (B1) with single-corruption-induced scenarios and Benchmark 2 (B2) with two-corruption-induced scenarios. The actual experimental results will be presented in Section 4. Here, we illustrate the concept of comparisons. First, the Model-Clean object detection model undergoes transfer learning without any corruption and is expected to have the lowest performance among the four models. Next is Model-B1, which utilizes filtered corruption types to generate the single-corruption training dataset for improved training. Based on the filtered corruption types, Model-B2 utilizes training data with two types of corruption to improve the model detection performance. Finally, for the Model-Original, which undergoes enhanced training using all types of corruption, significant training time is required. If the experimental results E-1-1 and E-2-1 are similar to E-A-1, it indicates the effectiveness of the filtering algorithm proposed in this study. This suggests that the algorithm can maintain good system robustness in a shorter training time. Given that real-world traffic situations may involve the simultaneous occurrence of two types of corruption, it is essential to investigate their impact on the model. Thus, in addition to conducting single-corruption-type testing and validation, it is essential to perform testing and validation on benchmark B2, which includes two corruption type combinations, to ensure that the model’s robustness meets system requirements.

Therefore, we will analyze whether Model-B1 and Model-Original, trained using only one type of corruption in the enhanced training dataset, can maintain good robustness performance in B2 with combinations of two types of corruption. Conversely, training the model with two combinations of corruption types may result in good performance in B2. However, it is yet to be determined whether it can also exhibit strong robustness performance in B1. The following section will offer additional analysis and discussion of the experimental results. Ultimately, we hope that these experimental results will help designers find a suitable solution by considering model testing and training time enhancements.

## 4. Experimental Results and Analysis

### 4.1. Corruption Types and Benchmark Dataset Generation

This work is based on the VTD vehicle simulation platform and showcases two of the most common driving scenarios: city roads and highways. Two city road scenarios and two highway scenarios were created. The video footage was recorded using cameras installed on the windshields of simulated vehicles, with a resolution of 800 × 600 pixels and a frame rate of 15 frames per second (FPS). These recordings were used to create benchmark test datasets and to train models for enhancement. In total, there were 5400 original, unaltered video frames for the city road scenarios and 3840 for the highway scenarios.

As indicated in Table 9, we employed the weather-related and noise-related generation tools proposed in this study to illustrate the robustness and performance testing of object detection models under nine different types of corruption. The types of inclement weather include fog and rain. There are five severity regions for fog, with visibility ranging from 200 to 20 m. In the event of heavy rain, there are two severity levels, with rainfall ranging from 43 to 54.8 mm/h, leading to a visibility of approximately 300 to 100 m. Noise corruption types include seven categories: hot pixel defect, single pixel defect, cluster 22/33/44 pixel defect, column pixel defect, and raindrop corruption. Among these, raindrop contamination is categorized into two severity levels, based on rainfall rates of 20–50 mm/h and raindrop diameters of 0.183–0.229 cm. The remaining types of noise corruption are categorized into three severity regions, and the level of image noise corruption ranges from 1% to 15%.

We started by generating two original, corruption-free image datasets using the VTD simulation platform: one for city scenes and the other for highways, each containing 5400 images. The benchmark testing dataset includes datasets for all individual corruption types and severity regions. For the complete benchmark testing dataset, we generated datasets with corruption for each corruption type and severity region by using weather and noise-related corruption generation tools on the original, corruption-free image dataset. Overall, we demonstrated a total of nine different corruption types, along with an image type with no corruption. As mentioned before, each severity region in a corruption type needs 5400 original, corruption-free images to create single-corruption-induced scenarios. Therefore, the total number of images for the city and highway original benchmark testing datasets is 5400 images × 28 = 151,200 images, respectively.

**Figure 11:**
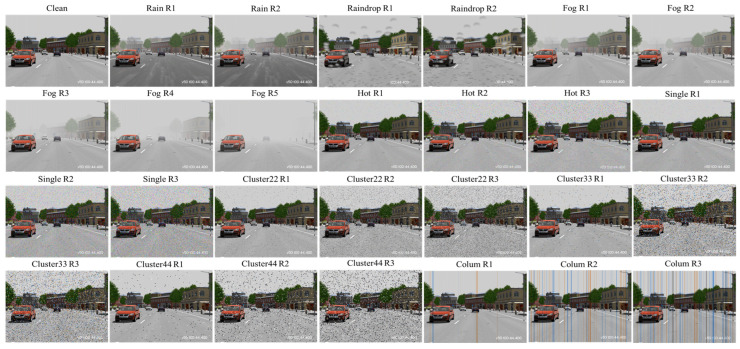
Comparison of the effects of corruption in regions of varying severity for nine types of corruption in weather and noise.

As displayed in Figure 11, using the VTD simulation combined with the weather and noise-related generation tools, we can clearly observe that as severity increases, the corruption in the images also significantly increases. Users can quantitatively adjust severity to generate benchmark testing image datasets with varying degrees of corruption. This capability helps improve test coverage for rare adverse weather or noise corruption scenarios, ensuring that the system’s reliability and robustness meet requirements.

### 4.2. Corruption Type Selection

The performance of object detection models in autonomous driving perception systems may vary due to the differences between city and highway scenarios. City areas are characterized by the presence of various buildings and traffic participants, in contrast to the relatively simpler highway environment. The reliability and robustness of these models may vary under different scenarios and types of corruption. Therefore, we present experimental demonstrations in both city and highway scenarios. We utilized the “faster_rcnn_resnet101_coco” pre-trained model available in the TensorFlow model zoo. We applied corruption filtering algorithms and adjusted various overlap threshold parameters to eliminate corruption types with high similarity. We then compared and analyzed the differences in types of corruption after filtering in both city and highway scenarios.

**Figure 12:**
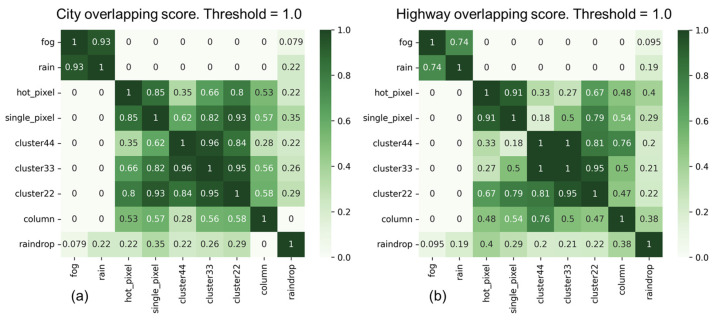
Overlapping score matrix for corruption types in city and highway scenarios. (**a**) City; (**b**) highway.

As shown in Figure 12, the experimental results of the overlapping scores for all types of corruption in city and highway scenarios indicate that two types of corruption, “fog” and “rain”, demonstrate high similarity in both scenarios. However, the similarity of these two types of corruption to other types of corruption is significantly lower. Furthermore, the “raindrops” corruption type also exhibits significant differences compared to other corruption types. In addition, there are noticeable differences in the overlapping scores between the two scenarios, particularly in the “noise” corruption category. We observed another interesting point: the overlapping score between the “raindrop” and “column” corruption types is 0.38 in the highway scenario, whereas it is zero in the city scenario. This variation may be primarily attributed to the traffic conditions in the city and on highways. Therefore, it is necessary to conduct separate testing and validation analyses for different categories of scenarios to assess the impact of various types of corruption and their severity.

**Figure 13:**
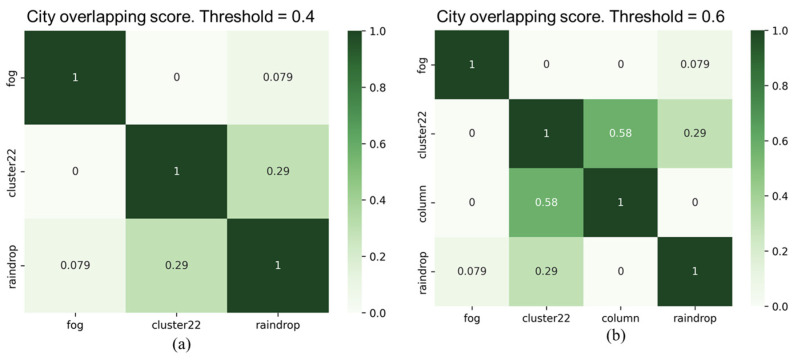
Filtered types of corruption in a city setting using various thresholds. (**a**) Threshold = 0.4; (**b**) threshold = 0.6.

**Figure 14:**
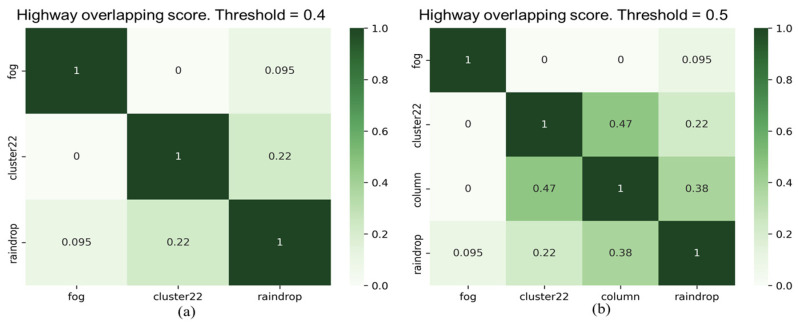
Filtered types of corruption in highway scenarios using various thresholds. (**a**) Threshold = 0.4; (**b**) threshold = 0.5.

Figure 13 and Figure 14 display filtered corruption types in city and highway scenarios with varying thresholds. For city settings, the thresholds were set at 0.4 and 0.6, leading to the identification of three and four types of corruption, respectively. For highway scenarios, the thresholds were 0.4 and 0.5, resulting in the identification of three and four filtered corruption types, respectively. In the city setting, using an overlap threshold of 0.6, the corruption types were reduced to four, namely “fog”, “cluster 22”, “raindrop”, and “column”. When the threshold was reduced to 0.4, column corruption was removed, while the other three types of corruption remained unchanged. On the other hand, in the highway scenario, we observed the same types of filtered corruption as in the city scenario when setting overlap thresholds at 0.4 and 0.5.

**Figure 15:**
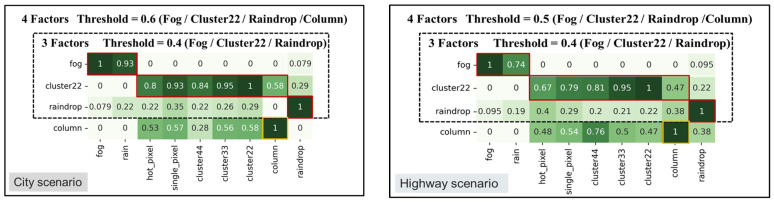
In urban and highway scenarios, corruption groups are formed by different thresholds. Additionally, in the corruption overlap score table, the darker the color, the higher the similarity between the two. A red box represents three types of corruption group, while four types of corruption group are additionally outlined with a yellow box.

After applying the filtering similarity corruption algorithm, the corruption grouping algorithm utilized the filtering results to obtain the corresponding corruption groups. Figure 15 shows the corruption groups formed at various thresholds in city and highway scenarios. For a threshold of 0.4, the first group consists of two types of corruption: “fog” and “rain”, with “fog” representing this group. The second group consists of six types of corruption: “hot pixel”, “single pixel”, “cluster 22/33/44”, and “column”, with “cluster 22” serving as the representative type of corruption. The third group consists of only one type of corruption, known as “raindrop”. For a city threshold of 0.6 and a highway threshold of 0.5, the only change occurs in the group represented by “cluster 22”, where the corruption in the “column pixel” becomes a separate group.

### 4.3. Model Corruption Type Vulnerability Analysis and Enhanced Training

If the object detection model is trained with less severe or almost non-impactful data, or if it uses a dataset that includes all possible corruption types and severity regions, it may consume a significant amount of training time and data space, with limited improvement in overall model robustness. Concerned about the effectiveness of the training process for the models, we propose an approach based on vulnerability analysis of corruption types to discover the region of severity that has the most significant impact on the accuracy of the model for the considered types of corruption. The primary goal of this approach is to identify the region of severity with the greatest impact on the model for each type of corruption. Subsequently, we can propose an enhanced training dataset tailored to the vulnerability region of each filtered corruption type and conduct transfer learning. This approach significantly reduces the size of the augmented training dataset and the time required for model training. At the same time, it efficiently enhances the model’s robustness against the identified types of corruption.

We will use the example shown in Table 10 to illustrate how to identify the vulnerability region for a corruption type. Table 10 lists nine types of corruption considered, denoted as C_1_~C_9_, and according to Figure 13b, C_1_ and C_7_~C_9_ are representative corruption types selected from the corruption filtering algorithm. In the “Fog” corruption type, visibility ranges from 200 to 20 m, with a 30 m difference between each severity level. This results in a total of seven different levels of severity (NSL). In the “Noise” corruption types, such as “Cluster 22” and “Column”, we establish severity levels ranging from 1% to 15%, with a 2% variance between each severity level, resulting in eight distinct levels of severity for each type. Finally, for the “Raindrop” corruption type, we vary the rain quantity from 20 to 50 mm and the raindrop diameter from 0.183 to 0.229 cm, resulting in three different levels of severity. Then, we create test datasets for the representative types of corruption with severity levels, as shown in Table 10, for both city and highway scenarios. Therefore, the number of test datasets required for C_1_ and C_7_~C_9_ are 7, 8, 8 and 3, respectively.

Next, we conducted the model test for each test dataset and analyzed the variations in slope of the different severity regions in relation to changes in object detection accuracy. The test results are presented in Table 11. As shown in Table 11, in this experiment, we utilized the faster_rcnn_resnet101_coco pre-trained model provided in the TensorFlow model zoo for object detection. Subsequently, we utilized two separate clean datasets: one representing city roads and the other representing highways. We first conducted transfer learning to develop two object detection models, namely the city_clean and highway_clean models. Next, we proceeded to assess and compare the effects of various types and levels of corruption on the city_clean and highway_clean models in the two different scenarios. From the experimental results, it is evident that although the same corruption types were tested in both city and highway scenes, the model performs significantly better in the highway scene. The primary reason for this difference is that the overall complexity of backgrounds in highway images is considerably lower compared to city road scenes. City roads are characterized by numerous buildings and diverse objects in the vicinity, which can lead to lower overall model detection accuracy. From Table 11, we can see notable variances in the object detection accuracy for the fog corruption type in both city and highway scenes. In the visibility range of 200 m to 20 m, the overall model’s Average Precision (AP) drops by 68.67% in city road scenes, whereas in the highway scene, the drop is only 49.06%. Specifically, at a severity level of 20 m, the test results show a difference of up to 32.56, emphasizing the more significant and severe impact of fog corruption on the model in city road scenarios.

Within the same type of corruption, we evaluated how different severity levels affected the model’s detection rate by analyzing the variation in detection accuracy across severity levels. For each type of corruption and its corresponding severity levels, we calculated the slope of the variation in model detection accuracy and identified the severity region with the most pronounced slope. This region represents the model’s vulnerability to a specific type of corruption, indicating the area where the model requires the most enhanced training. From Table 11, we observe that the visibility range between 50 m and 20 m indicates the severity zone with the steepest change in fog conditions, both in city and highway settings. For the other three types of corruption, their impact on object detection in city and highway scenes is less significant than that of fog. From the experimental results, it is evident that various types of corruption affect the model to different degrees. This insight enables us to identify which types of corruption require special attention in terms of enhancing the model’s detection capabilities. In city road environments, the areas of highest impact for Column and Cluster 22 corruption types are 13% to 15%, while for Raindrop corruption, they range from 20 mm to 35 mm/h. In the highway scenario, the severity range for Column corruption is 1% to 3%, while the chosen severity regions for other types of corruption are the same as those in the city scene. Subsequently, considering the identified vulnerability regions for different types of corruption, we propose using an enhanced training dataset to facilitate transfer learning and enhance the model’s robustness. This approach effectively enhances the model’s robustness while also reducing the training time.

The scene for the enhanced training dataset was constructed using the VTD simulation platform, which established city and highway scenarios. The simulation was set to generate 15 frames per second, resulting in a total of 3840 frames for each scenario. Additionally, weather conditions and image noise, along with a raindrop generation tool proposed in this research, were utilized to create training datasets for each type of corruption, including their respective vulnerability regions. Furthermore, within the dataset for model improvement and transfer learning, we divided it into two benchmarks: Benchmark 1, which focuses on training for enhancing a single type of corruption, and Benchmark 2, which combines training for two types of corruption. To prevent the problem of model forgetting during training, we included a set of clean images, unaffected by any corruption, in each dataset. This helped to ensure that the accuracy of object detection for objects unaffected by corruptions was not compromised.

We conducted a comparative analysis of the robustness performance in city and highway environments using various improved training datasets for model transfer learning. Based on the publicly available pre-trained model in the TensorFlow model zoo, we utilized the faster_rcnn_resnet101_coco model, which we will refer to as the M1 object detection model. The hyperparameters of the training process for transfer learning are depicted as follows. The number of input images per step (IS) was fixed at two, the total number of epochs (NE) for model enhancement transfer learning was set to 8, and the experiment was conducted on a computer equipped with an Intel i5-10400 CPU, DDR4 16 GB RAM, and a GeForce RTX™ 3060 GPU. The training time required for each step in the M1 model for transfer learning, denoted as ETM1, was approximately 0.275 s. We then compared the training times and the improvements in robustness across different training datasets.

Table 12 presents the performance of our detection model in city and highway scenarios after undergoing reinforcement training for a single-corruption type using four distinct reinforcement learning datasets. The following datasets are described below.

1. Dataset without any corruption.
2. Dataset containing all corruption types with all severity regions.
3. Dataset with three corruption types (F3) derived from the corruption filtering algorithm.
4. Dataset with four corruption types (F4) derived from the corruption filtering algorithm.

We examined how various reinforcement learning datasets affect the training time needed for model training, while keeping the same training hyperparameters as mentioned above. The relevant parameters are defined as follows:

- NEI: number of images contained in an enhancement dataset.
- NEC: number of enhancement corruption types.
- $EC_{i}$: the *i*-th enhancement corruption type, where *i* = 1 to NEC.
- $NER\left(EC_{i}\right)$: number of severity regions for enhancement corruption type *i*, where *i* is from 1 to NEC.
- $EC_{i}\left(R_{j}\right)$: the *j*-th region of enhancement corruption type *i*, where *j* = 1 to *NER(EC_i_*).
- For each severity region $EC_{i}\left(R_{j}\right)$, the number of reinforcement training images is denoted as NEI.
- IS: number of input images for each transfer learning step.
- ET: estimated time of required for transfer learning in second per step for object detection model.
- NE: the total number of epochs for model reinforcement transfer learning.
- MET: the training time required for object detection model reinforcement.

The first training model, M1-Clean, utilizes only the clean training dataset without any corruption for reinforcement training. Here, *NEI* consists of 3840 images. The estimated training time for the reinforcement model is calculated as $MET=\frac{NEI\times ET\times NE}{IS}$. Since this model is trained exclusively with a clean dataset and without any corruption, it requires the shortest time and the fewest training images. Therefore, M1-Clean serves as the baseline for evaluating the robustness of other reinforcement learning models.

The second training model, M1-All, incorporates all types of corruption and severity regions into the reinforcement training dataset. As shown in Table 10, there are a total of nine types of corruption (*NEC* = 9). *NER*(*EC_Fog_*) has seven severity levels, while both *NER*(*EC_Rain_*) and *NER*(*EC_Raindrop_*) have three severity levels each. Finally, there are six types of corruption: Hot, Single, Cluster 22/33/44, and Column, each with eight severity levels.

In total, there are 61 severity levels for all types of corruption. Therefore, the total number of images in the reinforcement training dataset, including the clean type, is {missing number}. The estimated training time for this model is $\frac{62\times NEI\times NE\times ET}{IS}$, making it the model with the highest number of reinforcement training images and the longest training time among the four models. The third and fourth training models, M1-B1F3 and M1-B1F4, were created using the improved training dataset derived from the vulnerable regions associated with representative corruption types. Both city and highway scenes exhibit the same set of representative corruption types, with a total of three corruption types (NEC = 3) for M1-B1F3 and four corruption types (NEC = 4) for M1-B1F4. Therefore, the estimated training times for M1-B1F3 and M1-B1F4 are $\frac{4\times NEI\times NE\times ET}{IS}$ and $\frac{5\times NEI\times NE\times ET}{IS}$, respectively. Overall, the required training time for M1-B1F3 and M1-B1F4 is significantly lower compared to M1-All.

As shown in Table 12, the four reinforcement training datasets not only vary significantly in the number of images but also exhibit a substantial difference in the estimated training time for model reinforcement transfer learning, particularly the M1-All model, which is trained with all types of corruption and severity levels. The overall training time for M1-All is much higher compared to the models with reduced corruption types (M1-B1F3 and M1-B1F4). It is important to note that indiscriminately increasing the number of training images without a proper selection strategy can result in a significant increase in training time. On the other hand, if the additional reinforcement training data consists of non-severe or less impactful datasets, it may result in significant training costs and time without substantial improvements in the robustness of the object detection model. The effectiveness of M1-B1F3 and M1-B1F4 compared to the M1-All model will be discussed in the following subsection.

### 4.4. Robustness Analysis of Enhanced Training Models

Table 13 and Table 14 display the experimental results mentioned in Table 6 earlier. Four different test datasets were used to assess robustness. The assessment method involved summing and averaging the average precision (AP) scores of individual corruption types. The initial test dataset includes all types of corruption, as well as the clean dataset. The models were tested individually for each type of corruption, and the AP scores were then summed and averaged. The results indicate that the object detection model trained with representative corruption types closely resembles the model trained with all types of corruption. In the city setting of Table 13, the two models demonstrate AP scores of 75.8% and 75.06%, with a difference of only 0.74%. Similarly, in the highway scenario, the two models have very similar average precision (AP) scores of 89.38% and 88.55%, with a difference of 0.83%. Table 14 presents similar results. This demonstrates that the corruption-filtered algorithm proposed in this study effectively reduces training time by eliminating highly similar types of corruption while maintaining high training coverage. As a result, it achieves comparable robustness to models trained with all types of corruption.

The second test dataset was created by combining representative corruption types with the clean dataset. To objectively evaluate the robustness performance of these test datasets, AP scores were calculated by multiplying the group’s representative corruption type’s AP score by the number of corruption types in that group and then summing and averaging the results. This method enables a fair comparison between the test datasets formed by representative types of corruption and the entire test dataset of all corruption types. As shown in Table 13, the results for the test datasets formed by representative corruption types and the test dataset for all corruption types are very similar for City_M1-B1F3. The same phenomenon is observed for highway_M1-B1F3. The statement indicates that a test dataset formed by representative corruption types maintains a similar test coverage to a test dataset with all corruption types. Furthermore, we have observed similar results in Table 14.

From the experimental results, it is evident that M1-All requires the longest training time compared to the other models. However, it may not have the highest AP scores across all test datasets. In fact, certain models with shorter training times exhibit slightly higher AP scores than M1-All on specific test datasets. The potential explanation is that simply increasing the amount of augmented training data does not guarantee improved robustness. Instead, it may lead to catastrophic forgetting in the model, where more training time is spent without significantly improving robustness.

Therefore, another interesting point to explore is the impact of setting overlap threshold values on the number of representative corruption types and the coverage of model training and testing. In both city and highway scenarios, the M1-B1F3 and M1-B1F4 models demonstrate highly similar robustness performances on all types of corruption in the test dataset. In this context, three representative types of corruption are sufficient to cover all the corruption types considered. Adding a fourth type of corruption, such as column corruption, does not yield any significant improvement. The third and fourth test datasets in Table 13 and Table 14 were generated by corrupting groups. These test datasets can be used to verify the effectiveness of the representative corruption type in representing its corresponding corruption group. The results for x_M1-B1Fy, where x represents City or Highway and y represents 3 or 4, and M1-All for the third and fourth test datasets demonstrate the effectiveness of the representative corruption type in representing its corresponding corruption group. For example, the data presented in the third test dataset of Table 13a are nearly identical, indicating that fog corruption can effectively represent rain corruption. Similarly, the data presented in the fourth test dataset indicate that cluster 22 effectively represents its corresponding corruption group. In summary, based on Table 13 and Table 14, we can conclude that fog and cluster 22 effectively represent their respective corruption groups.

### 4.5. Exploring Scenarios with Two Corruption Combinations

In real-world scenarios involving autonomous vehicles, they are often affected by multiple types of corruption simultaneously, such as rain and fog occurring together. Therefore, we should further investigate the model’s robustness performance in scenarios where two types of corruption are combined. To simplify the experiments, we utilized three representative types of corruption in city and highway scenarios to generate reinforcement training datasets for the M1-B2F3 model. As shown in Table 7 and Table 15, we divided the severity regions of corruption types into two sub-regions, denoted as SR1 and SR2. The SR1 and SR2 sub-regions are depicted in Table 15 with severity data, indicating the severity level of corruption types within each sub-region. There are three pairs of two-corruption combinations, including (Fog and Cluster 22), (Fog and Raindrop), and (Cluster 22 and Raindrop). To simplify the demonstration, the training datasets only consider the combinations of corruption type 1 (SR1) and corruption type 2 (SR1), as well as corruption type 1 (SR2) and corruption type 2 (SR2) for the two-corruption-type combinations, as shown in Table 15. However, we can also follow the combinations outlined in Table 7 to enhance the training coverage.

For each combination of two types of corruption, we generated its reinforcement training dataset using the following method: we used the original corruption-free image dataset. The first half of the images was generated with the SR1 severity combination from Table 15 to introduce scenarios induced by two corruptions, and the second half of the images was generated with the SR2 severity combination to introduce similar scenarios. For instance, in the case of the Fog and Cluster 22 combination, the first half of the image dataset was generated with a severity combination of 42.5 m + 13.5%, and the second half was created with a severity combination of 27.5 m + 14.5%. The training datasets for reinforcement learning for the two types of corruption required 4×NEI images, and the test datasets for two types of corruption were constructed in the same manner.

Table 16 presents the performance of models trained on single- and two-corruption types in city and highway scenarios, using single- and two-corruption test datasets. From this data, it can be observed that the performance of the model trained with two types of corruption is slightly inferior to the model trained with a single type of corruption on the B1 single-corruption test dataset, and it approximates the results of the M1-All model trained with all types of corruption. All four models exhibit a significant decrease in performance on the two-corruption test datasets, with the model trained using two corruption types outperforming the others. This suggests that in scenarios involving combinations of two types of corruption, despite their lower probability of occurrence, testing such combinations can reveal weaknesses in models trained to handle a single type of corruption. This highlights the need for specific training on scenarios involving two types of corruption to enhance the overall robustness of the model. This area could be explored in future research.

### 4.6. Real-World Scenario Testing and Verification

In real-world driving environments, autonomous vehicles’ perception systems may encounter adverse conditions or potential corruptions, which can lead to system failures or anomalies. To enhance the test coverage of adverse scenarios or corner cases in the aforementioned experiments, we created datasets that include different types of weather and image noise corruption using a vehicle simulation platform. Additionally, we developed tools for generating scenario-based test datasets. The results of the experiments demonstrate that by identifying model vulnerabilities through the vulnerability analysis proposed in this study and creating effective training datasets to enhance model vulnerability, the tolerance and robustness of object detection models to weather and noise-related corruptions can be significantly improved.

From the previous experimental results, it has been demonstrated that the proposed approach for robustness verification and enhancement can effectively improve the resilience of the detection models when tested against benchmark test datasets containing various types of corruption in simulated environments. To validate the performance of object detection models trained using our method in real-world scenarios, we utilized the DAWN real-world adverse weather driving scenario dataset [40] and the Foggy Cityscapes real-world scene dataset, which feature adverse weather conditions, as benchmark test datasets. These were used to test the performance of our trained models in real adverse driving scenarios. In the DAWN dataset, we excluded images with snowy corruption types that were not included in the experiments, as well as images with no vehicles. Additionally, for Foggy Cityscapes [41], we set the visibility parameter β to 0.08 to create denser fog, reducing visibility to approximately 37 m in real-world scene datasets. Then, we could assess the strength and dependability of models trained using simulated scenarios with these two real-world test datasets.

Table 17 presents the performance of four object detection models trained using various enhanced training datasets for city and highway scenarios. We observed that the robustness performance of the models in real-world scenarios is significantly lower compared to the results obtained in virtual simulation environments, which is a common phenomenon in current virtual simulation environments. It should be noted that the model’s performance significantly deteriorates when tested on the Foggy Cityscapes dataset due to the severe fog conditions under which the dataset was created. Our analysis primarily focuses on the verification and enhancement procedures for robustness proposed in this research, as well as the performance of the models trained using these methods in real-world scenarios. We observed that the models trained using a corruption-enhanced dataset indeed demonstrate better robustness in real-world scenarios compared to models that have not undergone corruption-enhanced training.

From Table 17, we can see that the robustness performance of the M1-B1F3 model on the real-world test dataset is higher in both city and highway scenarios compared to the M1-Clean model. This demonstrates that the approach proposed in this research is indeed capable of enhancing the resilience of object detection models. We also found that the models trained in city scenarios demonstrate better robustness performance compared to those trained in highway scenarios, with the exception of the M1-B1F3 model in the DAWN test dataset. This is likely because city environments are more complex, leading to more effective training.

Furthermore, we implemented a strategy based on the approach outlined in reference [42], which involves integrating a small amount of actual image data for subsequent transfer learning to further improve the models. This method has proven to be effective in improving the resilience of object detection models in real-world environments. Therefore, we introduced 800 images from the BDD dataset that were not affected by any corruption into both the M1-Clean and M1-B1F3 models. Subsequently, we conducted secondary transfer learning and validated the models using two real-world benchmark datasets that included adverse weather conditions. The experimental results clearly show that the M1-Clean_R800 and M1-B1F3_R800 models, which were trained with the inclusion of a small amount of actual data, can significantly enhance robustness compared to models trained solely on simulated datasets. Particularly, the M1-B1F3_R800 model demonstrates significant improvement in robustness performance under adverse real-world weather conditions compared to models trained solely on simulated datasets. The experimental results overall validate that the proposed robustness verification and enhancement procedures in this research can achieve high training and test coverage in both simulated and real-world environments for object detection models. Additionally, our approach can significantly reduce the time needed for training and validating model enhancements. Through the various model enhancement schemes presented, designers can choose a suitable method to improve object detection models and conduct efficient test verification.

## 5. Conclusions and Future Works

The design and testing of autonomous driving systems require demonstrating their reliability and safety in any environment, particularly when it comes to robustness testing and the verification of perception systems. Based on sensor-based perception systems, this study presents a comprehensive approach for validating and improving robustness using a simulation platform. We explore issues related to test scenario coverage and effectiveness, as well as scenario complexity, including training and testing dataset sizes and time efficiency. There are numerous factors that affect the safety of autonomous driving. In this study, we specifically focus on the impact of weather and noise on autonomous driving perception systems. We examine the effects of single-corruption- and two-corruption-induced scenarios on the robustness of these systems. Given the complexity of single corruption and combinations of multiple corruptions in test scenarios, as well as the substantial testing time required, it is essential to ensure the quality of test datasets and to conduct rapid testing and validation during the early stages of system development. Therefore, we propose an efficient corruption filtering algorithm to reduce the number of corruption types considered, in order to mitigate the complexity of test datasets while maintaining good test coverage. We are examining the correlation between the “overlap threshold” parameter in the corruption filtering algorithm and scenario complexity, time cost, and test scenario coverage. This parameter setting affects the number of selected corruption types, the size of training and testing datasets, training and testing time, and test coverage rate. Through this analysis, an appropriate “overlap threshold” parameter value can be determined to meet the requirements of effectiveness and efficiency. This will result in a set of optimal benchmark test datasets that satisfy time cost and test scenario coverage requirements. This ensures improved test scenario coverage and effectiveness in less time for testing.

To accelerate the creation of benchmark datasets, we have developed tools for generating simulated test scenarios. These tools include a weather-related test scenario generator and sensor noise injectors to emulate real traffic environments. We then use these benchmark datasets to evaluate object detection models for perception systems. A vulnerability analysis of the model was conducted to pinpoint susceptible areas to corruptions and develop training datasets that enhance the model’s ability to withstand weather- and noise-related corruptions. This, in turn, improves the robustness of the perception system. We utilize case studies to illustrate the process of generating test scenarios associated with weather conditions (e.g., rain, fog) and noise factors (e.g., camera pixel noise). Subsequently, we conduct robustness testing of the perception system by employing object detection models within these test scenario datasets. Then, the corruption similarity filtering algorithm is used to identify the types of corruption that would represent all the considered types of corruption. We demonstrate the identification of vulnerable regions for typical corruption types and the improvement process using data augmentation techniques to create effective training datasets for enhancing the resilience of the perception system. We will further discuss the impact of two-corruption-induced scenarios on the robustness of the models and leave this issue to be explored in future research. Finally, we validated our models trained in a simulated environment with real-world adverse test datasets to confirm the effectiveness of our proposed approach.

In addition to the weather- and noise-related corruptions proposed in this study, various lighting conditions also significantly impact robustness. However, under extremely adverse lighting conditions, robustness can be significantly reduced even without any additional corruption. Therefore, in this study, to simplify the analysis of the impact of corruption and severity on robustness, we did not include the effects of varying lighting conditions. In the future, we will integrate the analysis of the impact of different lighting conditions, backlighting, and sky conditions on robustness. The impact of combining multiple corruptions on robustness will also be further investigated. It is evident that the scenarios could involve multiple sources of pollution, such as fog and noise, occurring simultaneously. As shown in Table 16, this topic warrants further exploration in future research.

Furthermore, we will continue to optimize the testing and validation of various corruption combinations and enhance the robustness, thereby increasing the test coverage of autonomous driving systems in challenging environments or edge cases. We aim to propose more comprehensive test datasets related to corruptions to improve the reliability and robustness of autonomous driving technology, especially for model-in-the-loop, software-in-the-loop, and hardware-in-the-loop development environments. The toolset developed in this study will be integrated into the VTD scenario-based platform to support a model-based design process and establish a virtual self-driving test platform.

## Figures and Tables

**Table 1:** Definition of severity regions for various weather-related corruption types.

Type	No. Severity Region (R_n_)	R_1_	R_2_	R_3_	R_4_	R_5_
Fog Visibility (m)	5	200~164	164~128	128~92	92~56	56~20
Rain mm/h	2	43~48	48~55	-	-	-

**Table 2:** Subinterval definitions for severity regions of different weather-related corruption types.

Type	Severity Region	No. Subsections (STn)	ST1	ST2	ST3	ST4	ST5
Fog Visibility (m)	R_1_	5	200	191	182	173	164
Rain mm/h	R_1_	5	43	44.25	45.5	46.75	48

**Table 3:** Weather parameters.

Precipitation Type	Precipitation Intensity	Visibility (m)	Time of Day in Second	Cloud State
None, Rain, Snow	0.0~1.0	0~100,000	0~86,400	Off: Sky off/0: Blue sky/4: Cloudy/6: Overcast/8:Rainy

**Table 4:** Severity levels of hourly rainfall and their corresponding visibility.

Type	Number of Severity Region (R_n_)	R_1_	R_2_
Rain mm/h	2	43~48 mm/h	48~55 mm/h
		Visibility ≈ 300~200 m	Visibility ≈ 200~100 m

**Table 5:** Raindrop models and their corresponding raindrop volume size ranges.

Raindrop	2 µL	5 µL	10 µL	15 µL	20 µL
**Volume**	>0 & <5	≥5 & <10	≥10 & <15	≥15 & <20	≥20 & <91

**Table 6:** Comparative analysis of models trained on different datasets and tested on various testing sets.

	Original Corruption Type Test Dataset	Filtered Corruption Types Test Dataset	(Fog, Rain) Test Dataset	(Hot, Single, Cluster 22, 33, 44) Test Dataset
**Model trained by** **filtered corruption types**	**A**	**B**	**E**	**F**
**Model trained by** **original corruption types**	**C**	**D**	**G**	**H**

**Table 7:** Severity setting for subregions of the worst-performing region in single-corruption type detection.

City 2 Corruptions	Fog	Cluster 22
**Vulnerability** **region**	Visibility 50~20 m	Percentage 13~15%
**SR1**	(50 + 35)/2 = 42.5 m	(13 + 14)/2 = 13.5%
**SR2**	(35 + 20)/2 = 27.5 m	(14 + 15)/2 = 14.5%

**Table 8:** Detection performance of various trained models on benchmark testing datasets with single and combined corruption types.

	B1 (AP)	B2 (AP)
**Model-Clean**	E-0-1	E-0-2
**Model-B1** (**Single corruption**)	E-1-1	E-1-2
**Model-B2** (**Two corruptions**)	E-2-1	E-2-2
**Model-Original**	E-A-1	E-A-2

**Table 9:** Number of severity regions and descriptions of severity levels for corruption types.

Type	Corruption Type	NER	Factor	R_1_	R_2_	R_3_	R_4_	R_5_
Clean	C_0_	1	Clean	Non-corruption				
Weather Relative (WR)	C_1_	5	Fog Visibility (m)	200~164	164~128	128~92	92~56	56~20
	C_2_	2	Rain	43~48.7 mm/h Vis.: 300 m−200 m	48.7~54.8 mm/h Vis.: 200 m−100 m	−	−	−
Noise Relative (NR)	C_3_	3	Hot	1~5%	5~10%	10~15%	−	−
	C_4_	3	Single	1~5%	5~10%	10~15%	−	−
	C_5_	3	Cluster 44	1~5%	5~10%	10~15%	−	−
	C_6_	3	Cluster 33	1~5%	5~10%	10~15%	−	−
	C_7_	3	Cluster 22	1~5%	5~10%	10~15%	−	−
	C_8_	3	Column	1~5%	5~10%	10~15%	−	−
	C_9_	2	Raindrop	20~35 mm/h D 0.183~0.207 cm	35~50 mm/h D 0.207~0.229 cm	−	−	−

**Table 10:** Test datasets were generated to simulate corruption types at various severity levels.

Type	Corruption Type	NSL	Factor	Severity Level
**Clean**		1	Clean	Non-corruption
**Weather Relative** (**WR**)	**C_1_**	**7**	**Fog**	**20 m, 50 m, 80 m, 110 m, 140 m, 170 m, 200 m**
	C_2_	3	Rain	43 mm/h, 48.7 mm/h, 54.8 mm/h
**Noise** **Relative** (**NR**)	C_3_	8	Hot	1%, 3%, 5%, 7%, 9%, 11%, 13%, 15%
	C_4_	8	Single	1%, 3%, 5%, 7%, 9%, 11%, 13%, 15%
	C_5_	8	Cluster 44	1%, 3%, 5%, 7%, 9%, 11%, 13%, 15%
	C_6_	8	Cluster 33	1%, 3%, 5%, 7%, 9%, 11%, 13%, 15%
	**C_7_**	**8**	**Cluster 22**	**1%, 3%, 5%, 7%, 9%, 11%, 13%, 15%**
	**C_8_**	**8**	**Column**	**1%, 3%, 5%, 7%, 9%, 11%, 13%, 15%**
	**C_9_**	**3**	**Raindrop**	**20 mm_d0183, 35 mm_d0207, 50 mm_d0229**

**Table 11:** Test results for city and highway scenes.

Fog			Column			Cluster 22			Raindrop		
Visibility	AP50	Slope	Percent	AP50	Slope	Percent	AP50	Slope	Precipitation	AP50	Slope
**City Scenario**											
200 m	75.53	-	1%	77.99	-	1%	77.79	-	20 mm/h	66.85	-
170 m	73.93	−0.05	2%	77.11	−0.44	2%	76.70	−0.54	35 mm/h	63.41	−0.229
140 m	70.49	−0.11	5%	75.22	−0.95	5%	75.47	−0.62	50 mm/h	61.11	−0.153
110 m	64.30	−0.21	7%	74.10	−0.56	7%	73.26	−1.10			
80 m	56.98	−0.24	9%	73.92	−0.09	9%	70.28	−1.49			
50 m	43.66	−0.44	11%	72.91	−0.50	11%	67.18	−1.55			
20 m	6.86	−1.23	13%	72.34	−0.28	13%	62.57	−2.31	**Clean**		
			15%	69.80	−1.27	15%	56.07	−3.25	78.27		
**Highway Scenario**											
200 m	88.48	-	1%	92.74	-	1%	93.28	-	20 mm/h	83.43	-
170 m	84.39	−0.14	2%	90.52	−1.11	2%	90.99	−1.15	35 mm/h	82.12	−0.087
140 m	82.28	−0.07	5%	90.54	0.01	5%	88.64	−1.17	50 mm/h	81.06	−0.071
110 m	77.29	−0.17	7%	89.94	−0.30	7%	86.44	−1.10			
80 m	69.73	−0.25	9%	88.00	−0.97	9%	84.09	−1.18			
50 m	58.83	−0.36	11%	87.31	−0.34	11%	81.75	−1.17			
20 m	39.42	−0.65	13%	87.11	−0.10	13%	79.41	−1.17	**Clean**		
			15%	86.19	−0.46	15%	74.40	−2.50	93.11		

**Table 12:** Number of images and training time required for model reinforcement training on city and highway scenarios.

**City & Highway** **M1-Clean**	**Clean**					**Total Images**	**Training** **Time (hours)**
						3840	1.173
**City & Highway** **M1-All**	**Clean, Fog, Rain, Hot, Single, Cluster 44,** **Cluster 33, Cluster 22, Column, Raindrop** (**All Severity Range**)					238,080	72.75
**City_M1-B1F3 & Highway_M1-B1F3** **(Threshold 0.4/3 Corruptions)**	**Clean**	**Fog**	**Cluster 22**	**Raindrop**		15,360	4.69
		Visibility 50~20 m	Percentage 13~15%	Rainfall: 20~35 mm/h Raindrop diameter: 0.183~0.207 cm			
**City_M1-B1F4** **(Threshold 0.6/4 Corruptions)**	**Clean**	**Fog**	**Cluster 22**	**Raindrop**	**Column**	19,200	5.87
		Visibility 50~20 m	Percentage 13~15%	Rainfall: 20~35 mm/h Raindrop diameter: 0.183~0.207 cm	Percentage 13~15%		
**Highway_M1-B1F4** **(Threshold 0.5/4 Corruptions)**	**Clean**	**Fog**	**Cluster 22**	**Raindrop**	**Column**	19,200	5.87
		Visibility 50~20 m	Percentage 13~15%	Rainfall: 20~35 mm/h Raindrop diameter: 0.183~0.207 cm	Percentage 1~3%		

**Table 13:** Comparison of the performance of three representative models for training on different types of corruption using various test datasets. (**a**) city model and scenario; (**b**) highway model and scenario.

		All Corruption Type Test Dataset	Test Dataset (Clean, Fog, Cluster 22, Raindrop)	Test Dataset (Clean, Fog, Rain)	Test Dataset (Hot, Single, Cluster 22/33/44)
(**a**)	**City_M1-B1F3**	75.80	75.24	72.51	77.41
	**City_M1-All**	75.06	74.14	72.52	76.38
(**b**)	**Highway_M1-B1F3**	89.38	89.64	89.74	90.03
	**Highway_M1-All**	88.55	88.48	89.19	89.22

**Table 14:** Performance of four representative corruption type training models on various test datasets. (**a**) City model and scenario; (**b**) highway model and scenario.

		All Corruption Type Test Dataset	Test Dataset (Clean, Fog, Cluster 22, Raindrop, Column)	Test Dataset (Clean, Fog, Rain)	Test Dataset (Hot, Single, Cluster 22/33/44)
(**a**)	**City_M1-B1F4**	**75.81**	**74.74**	**72.47**	**77.49**
	**City_M1-All**	**75.06**	**74.11**	**72.52**	**76.38**
(**b**)	**Highway_M1-B1F4**	**89.54**	**89.89**	**89.42**	**90.41**
	**Highway_M1-All**	**88.55**	**88.55**	**89.19**	**89.22**

**Table 15:** Examples of two combinations of corruption types.

**City_M1-B2F3 & Highway_M1-B2F3** **(Threshold 0.4/3 Factors)**	**Clean**		**Fog**		**Cluster 22**	**Raindrop**
			Visibility 50~20 m		Percentage 13~15%	Rainfall: 20~35 mm/h
SR1	**−**		42.5 m		13.5%	23.8 mm/h
SR2	**−**		27.5 m		14.5%	31.3 mm/h
Clean	Fog & Cluster 22		Fog & Raindrop		Cluster 22 & Raindrop	
	(42.5 m, 13.5%)	(27.5 m, 14.5%)	(42.5 m, 23.8 mm/h)	(27.5 m, 31.3 mm/h)	(13.5%, 23.8 mm/h)	(14.5%, 31.3 mm/h)

**Table 16:** Performance of single- and two-corruption-type trained models in city and highway scenarios on single- and two-corruption test datasets. (**a**) City environment; (**b**) highway environment.

		B1 Single Factor Test Dataset (Clean, Fog, Cluster 22, Raindrop)	B2 Two Factors Test Dataset (Clean, Fog & Cluster 22, Fog & Raindrop, Cluster 22 & Raindrop)
(**a**)	**City_M1-Clean**	68.34	40.78
	**City_M1-All**	74.14	57.00
	**City_M1-B1F3** (**Single corruption**)	75.24	54.67
	**City_M1-B2F3** (**Two corruptions**)	74.05	59.70
(**b**)	**Highway_M1-Clean**	82.66	61.43
	**Highway_M1-All**	88.48	69.20
	**Highway_M1-B1F3** (**Single corruption**)	89.64	67.48
	**Highway_M1-B2F3** (**Two corruptions**)	86.11	72.29

**Table 17:** Performance of models trained by simulation scenarios tested on real-world adverse test datasets. (**a**) City model; (**b**) highway model.

	(a)				(b)			
	City_ M1–Clean	City_ M1–B1F3	City_M1- Clean_R800_ Daytime Clear	City_M1- B1F3_R800_ Daytime Clear	Highway_ M1-Clean	Highway_M1-B1F3	Highway_ M1-Clean_R800_ Daytime Clear	Highway_ M1-B1F3_R800_ Daytime Clear
**DAWN** **Foggy/haze/mist/** **rain–storm** **(total 491 images)**	44.22	45.77	52.76	56.64	43	46.75	50.3	54
**Foggy Cityscapes** **Beta_008** **(total 2831 images)**	14.21	15.33	16.01	18.09	11.36	14.04	15.25	17.29

## Data Availability

Data are contained within the article.

## References

[1] Min K., Han S., Lee D., Choi D., Sung K., Choi J. SAE Level 3 Autonomous Driving Technology of the ETRI. Proceedings of the 2019 International Conference on Information and Communication Technology Convergence (ICTC).

[2] Klück F., Zimmermann M., Wotawa F., Nica M. Genetic Algorithm-Based Test Parameter Optimization for ADAS System Testing. Proceedings of the 2019 IEEE 19th International Conference on Software Quality, Reliability and Security (QRS).

[3] Koopman P., Wagner M. (2017). Autonomous Vehicle Safety: An Interdisciplinary Challenge. IEEE Intell. Transp. Syst. Mag..

[4] Pezzementi Z., Tabor T., Yim S., Chang J.K., Drozd B., Guttendorf D., Wagner M., Koopman P. Putting Image Manipulations in Context: Robustness Testing for Safe Perception. Proceedings of the 2018 IEEE International Symposium on Safety, Security, and Rescue Robotics (SSRR).

[5] Bolte J., Bar A., Lipinski D., Fingscheidt T. Towards Corner Case Detection for Autonomous Driving. Proceedings of the 2019 IEEE Intelligent Vehicles Symposium (IV).

[6] VIRES Simulationstechnologie GmbH (2022). VTD—VIRES Virtual Test Drive. https://www.embeddedindia.com/download/hexagon-vtd.PDF.

[7] CarMaker|IPG Automotive. https://ipg-automotive.com/en/products-solutions/software/carmaker/.

[8] Dosovitskiy A., Ros G., Codevilla F., Lopez A., Koltun V. CARLA: An Open Urban Driving Simulator. Proceedings of the 1st Annual Conference on Robot Learning.

[9] International Organization for Standardization (2018). Road Vehicles–Functional Safety.

[10] von Bernuth A., Volk G., Bringmann O. Simulating Photo-Realistic Snow and Fog on Existing Images for Enhanced CNN Training and Evaluation. Proceedings of the 2019 IEEE Intelligent Transportation Systems Conference (ITSC).

[11] Caesar H., Bankiti V., Lang A.H., Vora S., Liong V.E., Xu Q., Krishnan A., Pan Y., Baldan G., Beijbom O. nuScenes: A Multimodal Dataset for Autonomous Driving. Proceedings of the 2020 IEEE/CVF Conference on Computer Vision and Pattern Recognition (CVPR).

[12] Yu F., Chen H., Wang X., Xian W., Chen Y., Liu F., Madhavan V., Darrell T. BDD100K: A Diverse Driving Dataset for Heterogeneous Multitask Learning. Proceedings of the 2020 IEEE/CVF Conference on Computer Vision and Pattern Recognition (CVPR).

[13] Cordts M., Omran M., Ramos S., Rehfeld T., Enzweiler M., Benenson R., Franke U., Roth S., Schiele B. (2016). The Cityscapes Dataset for Semantic Urban Scene Understanding. arXiv.

[14] Geiger A., Lenz P., Urtasun R. Are We Ready for Autonomous Driving? The KITTI Vision Benchmark Suite. Proceedings of the 2012 IEEE Conference on Computer Vision and Pattern Recognition.

[15] Su J., Vargas D.V., Kouichi S. (2019). One Pixel Attack for Fooling Deep Neural Networks. IEEE Trans. Evol. Comput..

[16] Zofka M.R., Klemm S., Kuhnt F., Schamm T., Zöllner J.M. Testing and Validating High Level Components for Automated Driving: Simulation Framework for Traffic Scenarios. Proceedings of the 2016 IEEE Intelligent Vehicles Symposium (IV).

[17] Yu H., Li X. Intelligent Corner Synthesis via Cycle-Consistent Generative Adversarial Networks for Efficient Validation of Autonomous Driving Systems. Proceedings of the 2018 23rd Asia and South Pacific Design Automation Conference (ASP-DAC).

[18] Muckenhuber S., Holzer H., Rübsam J., Stettinger G. Object-Based Sensor Model for Virtual Testing of ADAS/AD Functions. Proceedings of the 2019 IEEE International Conference on Connected Vehicles and Expo (ICCVE).

[19] Menzel T., Bagschik G., Isensee L., Schomburg A., Maurer M. From Functional to Logical Scenarios: Detailing a Keyword-Based Scenario Description for Execution in a Simulation Environment. Proceedings of the 2019 IEEE Intelligent Vehicles Symposium (IV).

[20] Zhu H., Ng M.K. (2020). Structured Dictionary Learning for Image Denoising Under Mixed Gaussian and Impulse Noise. IEEE Trans. Image Process..

[21] Wu D., Du X., Wang K. An Effective Approach for Underwater Sonar Image Denoising Based on Sparse Representation. Proceedings of the 2018 IEEE 3rd International Conference on Image, Vision and Computing (ICIVC).

[22] Wang Z., Bovik A.C., Sheikh H.R., Simoncelli E.P. (2004). Image Quality Assessment: From Error Visibility to Structural Similarity. IEEE Trans. Image Process..

[23] Horé A., Ziou D. Image Quality Metrics: PSNR vs. SSIM. Proceedings of the 2010 20th International Conference on Pattern Recognition.

[24] Cord A., Gimonet N. (2014). Detecting Unfocused Raindrops: In-Vehicle Multipurpose Cameras. IEEE Robot. Autom. Mag..

[25] Qian R., Tan R.T., Yang W., Su J., Liu J. (2018). Attentive Generative Adversarial Network for Raindrop Removal from a Single Image. arXiv.

[26] von Bernuth A., Volk G., Bringmann O. Rendering Physically Correct Raindrops on Windshields for Robustness Verification of Camera-Based Object Recognition. Proceedings of the 2018 IEEE Intelligent Vehicles Symposium (IV).

[27] Porav H., Musat V.-N., Bruls T., Newman P. (2020). Rainy Screens: Collecting Rainy Datasets, Indoors. arXiv.

[28] Deter D., Wang C., Cook A., Perry N.K. (2021). Simulating the Autonomous Future: A Look at Virtual Vehicle Environments and How to Validate Simulation Using Public Data Sets. IEEE Signal Process. Mag..

[29] Johnson-Roberson M., Barto C., Mehta R., Sridhar S.N., Rosaen K., Vasudevan R. Driving in the Matrix: Can Virtual Worlds Replace Human-Generated Annotations for Real World Tasks?. Proceedings of the 2017 IEEE International Conference on Robotics and Automation (ICRA).

[30] Gaidon A., Wang Q., Cabon Y., Vig E. VirtualWorlds as Proxy for Multi-Object Tracking Analysis. Proceedings of the 2016 IEEE Conference on Computer Vision and Pattern Recognition (CVPR).

[31] Jin J., Fatemi A., Pinto Lira W.M., Yu F., Leng B., Ma R., Mahdavi-Amiri A., Zhang H. RaidaR: A Rich Annotated Image Dataset of Rainy Street Scenes. Proceedings of the 2021 IEEE/CVF International Conference on Computer Vision Workshops (ICCVW).

[32] Hendrycks D., Dietterich T. (2019). Benchmarking Neural Network Robustness to Common Corruptions and Perturbations. arXiv.

[33] Muhammad K., Ullah A., Lloret J., Ser J.D., de Albuquerque V.H.C. (2021). Deep Learning for Safe Autonomous Driving: Current Challenges and Future Directions. IEEE Trans. Intell. Transp. Syst..

[34] Nowruzi F.E., Kapoor P., Kolhatkar D., Hassanat F.A., Laganiere R., Rebut J. (2019). How Much Real Data Do We Actually Need: Analyzing Object Detection Performance Using Synthetic and Real Data. arXiv.

[35] Marzuki M., Randeu W.L., Schönhuber M., Bringi V.N., Kozu T., Shimomai T. (2010). Raindrop Size Distribution Parameters of Distrometer Data With Different Bin Sizes. IEEE Trans. Geosci. Remote Sens..

[36] Serio M.A., Carollo F.G., Ferro V. (2019). Raindrop Size Distribution and Terminal Velocity for Rainfall Erosivity Studies. A Review. J. Hydrol..

[37] Roser M., Kurz J., Geiger A., Koch R., Huang F. (2011). Realistic Modeling of Water Droplets for Monocular Adherent Raindrop Recognition Using Bézier Curves. Proceedings of the Computer Vision—ACCV 2010 Workshops.

[38] Huang W., Lv Y., Chen L., Zhu F. Accelerate the Autonomous Vehicles Reliability Testing in Parallel Paradigm. Proceedings of the 2017 IEEE 20th International Conference on Intelligent Transportation Systems (ITSC).

[39] Laugros A., Caplier A., Ospici M. Using the Overlapping Score to Improve Corruption Benchmarks. Proceedings of the 2021 IEEE International Conference on Image Processing (ICIP).

[40] Kenk M.A., Hassaballah M. (2020). DAWN: Vehicle Detection in Adverse Weather Nature Dataset. arXiv.

[41] Sakaridis C., Dai D., Van Gool L. (2018). Semantic Foggy Scene Understanding with Synthetic Data. Int. J. Comput. Vis..

[42] Poucin F., Kraus A., Simon M. Boosting Instance Segmentation with Synthetic Data: A Study to Overcome the Limits of Real World Data Sets. Proceedings of the IEEE/CVF International Conference on Computer Vision.

